# Differences in Reactivation of Tuberculosis Induced from Anti-TNF Treatments Are Based on Bioavailability in Granulomatous Tissue

**DOI:** 10.1371/journal.pcbi.0030194

**Published:** 2007-10-19

**Authors:** Simeone Marino, Dhruv Sud, Hillarie Plessner, Philana Ling Lin, John Chan, JoAnne L Flynn, Denise E Kirschner

**Affiliations:** 1 Department of Microbiology and Immunology, University of Michigan Medical School, Ann Arbor, Michigan, United States of America; 2 Department of Biomedical Engineering, College of Engineering, University of Michigan, Ann Arbor, Michigan, United States of America; 3 Department of Molecular Genetics and Biochemistry, University of Pittsburgh School of Medicine, Pittsburgh, Pennsylvania, United States of America; 4 Department of Pediatrics, Division of Pediatric Infectious Disease, Children's Hospital of Pittsburgh, Pittsburgh, Pennsylvania, United States of America; 5 Department of Medicine, Albert Einstein College of Medicine, New York, New York, United States of America; 6 Department of Microbiology, Albert Einstein College of Medicine, New York, New York, United States of America; 7 Department of Immunology, Albert Einstein College of Medicine, New York, New York, United States of America; University of California Irvine, United States of America

## Abstract

The immune response to Mycobacterium tuberculosis (Mtb) infection is complex. Experimental evidence has revealed that tumor necrosis factor (TNF) plays a major role in host defense against Mtb in both active and latent phases of infection. TNF-neutralizing drugs used to treat inflammatory disorders have been reported to increase the risk of tuberculosis (TB), in accordance with animal studies. The present study takes a computational approach toward characterizing the role of TNF in protection against the tubercle bacillus in both active and latent infection. We extend our previous mathematical models to investigate the roles and production of soluble (sTNF) and transmembrane TNF (tmTNF). We analyze effects of anti-TNF therapy in virtual clinical trials (VCTs) by simulating two of the most commonly used therapies, anti-TNF antibody and TNF receptor fusion, predicting mechanisms that explain observed differences in TB reactivation rates. The major findings from this study are that bioavailability of TNF following anti-TNF therapy is the primary factor for causing reactivation of latent infection and that sTNF—even at very low levels—is essential for control of infection. Using a mathematical model, it is possible to distinguish mechanisms of action of the anti-TNF treatments and gain insights into the role of TNF in TB control and pathology. Our study suggests that a TNF-modulating agent could be developed that could balance the requirement for reduction of inflammation with the necessity to maintain resistance to infection and microbial diseases. Alternatively, the dose and timing of anti-TNF therapy could be modified. Anti-TNF therapy will likely lead to numerous incidents of primary TB if used in areas where exposure is likely.

## Author Summary

Tuberculosis (TB) is the leading cause of death due to infectious disease in the world today. It is estimated that 2 billion people are currently infected, and although most people have latent infection, reactivation occurs due to factors such as HIV-1 and aging. Antibiotic treatments exist; however, there is still no cure and the current vaccine has proven to be unreliable. Experimental science has uncovered a plethora of immune factors that help the host control infection and maintain latency. One such factor, tumor necrosis factor alpha (TNF), is a protein that facilitates cell–cell communication during an inflammatory immune response. Animal models have shown that TNF is necessary for control of TB infection. Different types of anti-TNF drugs were developed for patients with non-TB related inflammatory diseases such as rheumatoid arthritis and Crohn's disease. Some of these patients who had latent TB suffered reactivation, especially with one drug type. Because these studies cannot be performed in the mouse, and nonhuman primates are expensive, we developed a computational model to perform virtual clinical trials (VCTs) that predicted why reactivation occurs and why it happens differentially between the two classes of drugs tested. We make recommendations on how this issue can be combated.

## Introduction

Control of Mycobacterium tuberculosis (Mtb) infection is a result of a successful immune response that requires priming and activation of antigen-specific CD4+ and CD8+ T lymphocytes, recruitment of cells to the infection site (typically the lung), and production of cytokines, some of whose role is to activate macrophages. This leads to inhibition or killing of some but not all bacilli. Immunological structures (granulomas) form in the lung in response to persistent antigen and cytokine and chemokine signals. In 95% of infected hosts, M. tuberculosis (Mtb) persists without causing symptoms or disease. Latent infection can subsequently reactivate to cause active TB. Experimental evidence has revealed that tumor necrosis factor (TNF) plays a major role in host defense against Mtb in both the active and chronic phases of infection [1–4].

TNF action increases the phagocytosis by macrophages and enhances mycobacterial killing in concert with IFN-γ [3,5]. TNF is crucial in recruitment of inflammatory cells, stimulating chemokine production [6] and inducing adhesion molecules on vascular endothelium [7].

Table S1 summarizes data regarding TNF in Mtb murine models. TNF is a crucial component of both antibacterial protection and the inflammatory immune response. TNF-deficient mice exhibit disorganized granulomas, altered tissue pathology, high bacterial loads, and reduced survival [2,3]. TNF also possesses tissue-injuring properties that manifest in clinical settings including inflammation, auto-immune diseases, and transplant rejections [8,9]. In TB patients, peripheral increases in TNF have been implicated in clinical worsening [10]. In the absence of TNF signaling, disruption of granulomatous formation as well as dissolution of granulomas during chronic infection occurred, resulting in death of the mice [1,4,11].

TNF is initially a transmembrane (tmTNF) protein that undergoes cleavage by the specific metalloproteinase TNF-converting enzyme (TACE) to form a soluble trimer [soluble tumor necrosis factor (sTNF)] [12]. Both forms of TNF function by binding to one of two receptors, TNFR1 (TNFRp55) and TNFR2 (TNFRp75) [13]. It was reasoned that transmembrane ligands of TNF superfamily might elicit bidirectional signals (reverse signaling) [14]. That hypothesis was supported by data describing potential receptor-like properties of tmTNF [15]. The majority of reverse signaling described in lymphocytes is stimulatory [16,17], whereas monocytes are mainly inhibited in their effector functions [18–20]. For further details, we review TNF biology (sTNF and tmTNF, receptors, reverse signaling, and the role of lymphotoxin) in Text S1. Known effects of sTNF and tmTNF on macrophages and T cells are summarized in Table 1.

**Table 1:**
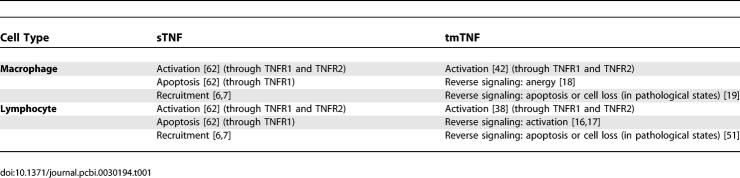
sTNF and tmTNF Effects on Lymphocytes and Monocytes/Macrophages

Several biologic inhibitors (antibodies and receptor fusion molecules) have been developed that interfere with TNF activity and are used to control inflammation in diseases such as rheumatoid arthritis [21,22] and Crohn's disease [23,24]. The importance of TNF in control of TB is highlighted by an increased susceptibility of these patients to TB reactivation [25,26]. The incidence of TB in individuals receiving antibody appears to be higher than in those treated with receptor fusion molecule [27], suggesting that there are differences in the mode of action of these agents. Alternatively, the dose and timing of anti-TNF therapy could be modified.

The present study takes a theoretical approach toward characterizing the role of TNF in protection against the tubercle bacillus in both active and latent infection. We extend our previous models [28–31] to investigate the roles and production of sTNF versus tmTNF. To explore the effects of TNF blockade, we study two anti-TNF agents, a TNF-neutralizing antibody and a soluble p75 TNF receptor fusion (TNFR2Fc). Using a mathematical model, it is possible to distinguish mechanisms of action of the anti-TNF treatments and gain insights into the role of TNF in TB control and pathology.

## Results

We describe results in these different areas of TNF study: mathematical modeling of typical infection progressions in humans, mechanisms driving infection outcomes, and anti-TNF therapies. Deletion and depletion experiments are discussed in Methods. Unless otherwise specified, all plots are on a linear-log scale.

**Figure 1:**
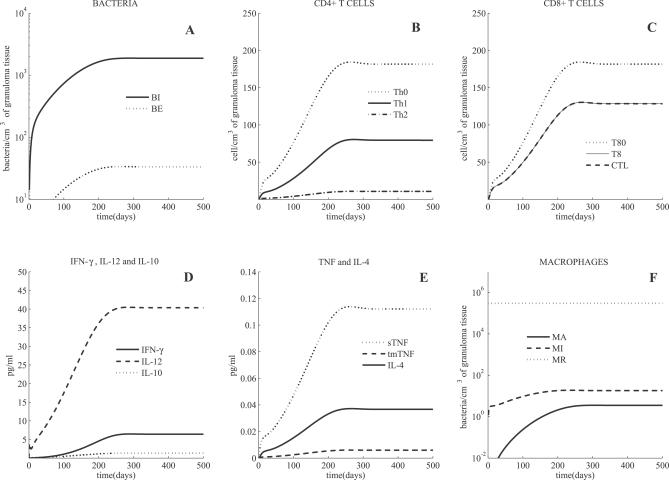
Mathematical Model Simulation of a Latent State Shown are intracellular and extracellular bacterial loads (A), CD4+ and CD8+ T cells (B,C) (linear scale), cytokines (D,E) (linear scale), and macrophages (F). The volumetric unit for cell and bacteria populations is number per cm^3^ of a granulomatous tissue. The unit of measure for cytokine concentrations is pg/mL of granuloma homogenate. BE, extracellular bacteria; BI, intracellular bacteria; MA, activated Mφ; MI, infected Mφ; MR, resident Mφ.

**Figure 2:**
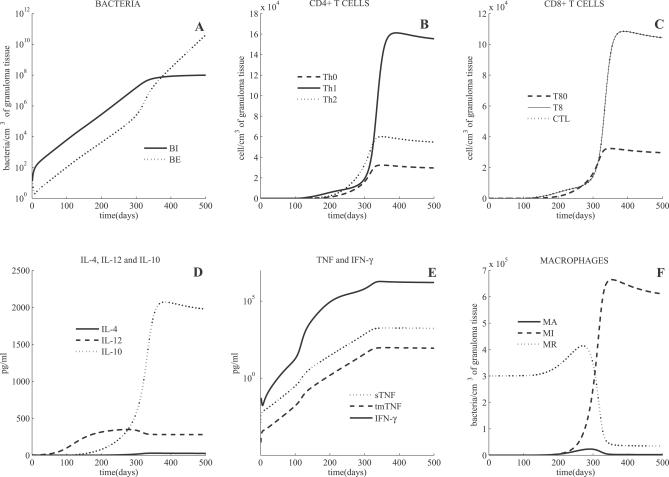
Mathematical Model Simulation of an Active TB State Shown are intracellular and extracellular bacterial loads (A), CD4+ and CD8+ T cells (B,C) (linear scale), cytokines (D) (linear scale) and (E) (linear-log scale), and macrophages (F) (linear scale). See Figure 1 for measure units and abbreviations. The main differences in parameter value choices used to distinguish active TB from latency in this simulation are the following: decreased lymphocyte TNF-dependent recruitment, increased macrophage TNF-dependent and independent recruitment, decreased CTL killing (k_52_), and increased extracellular bacteria growth rate (α_20_).

Negative control simulations have been performed on the model [31]. The model simulates both latent infection and active TB outcomes, depending on parameter values. Cell and cytokine profiles associated with latent and active TB are shown in Figures 1 and 2, respectively. As discussed previously [31], we use bacterial load as a marker of disease progression, where uncontrolled growth is indicative of active TB.

### Latent TB

Simulations predict that with an inoculum of 25 mycobateria [32], latency is achieved (i.e., bacterial numbers are controlled) in fewer than 300 days, under appropriate immunologic conditions. Latent TB is characterized by low levels of extracellular bacterial load (<50 bacteria per cm^3^ of granulomatous tissue), and all intracellular bacteria (Figure 1A) reside within a small number of chronically infected macrophages (MIs) (approximately 15 MI, with 50 intracellular bacteria each). The total population of T cells in latency (CD4+ and CD8+ T cells combined, Figure 1B and 1C) is comparable with numbers found experimentally, with a ratio of CD4+/CD8+ T cells approximately one, consistent with experimental observations [33,34]. During latency, TNF levels (Figure 1E) are on the order of 0.12 pg/mL (limited inflammation) as levels of MIs and MAs (activated microphages) are relatively low (and these are major TNF producers). This small amount of TNF is significant, as neutralizing this concentration of TNF drives the system into active TB (see the section “TNF depletion and anti-TNF treatments”). This indicates a critical role for even small amounts of TNF in maintaining latency. Predicted ranges for IFN-γ and IL-10 (Figure 1D) all correlate with studies measuring cytokine levels at the infection site [35–37]. Total macrophage numbers do not change significantly in the first year post-infection, and resident macrophages remain relatively constant, while numbers of MIs and MAs remain below 50 (Figure 1F).

### Sources of TNF during Latency

The roles played by different cellular sources of TNF involved in protective immunity remain unclear. During latency we evaluate and compare production of TNF by macrophages and lymphocytes (Table S2). The model predicts that macrophages are the main producers of TNF during the early phase of infection, and that once latency is achieved lymphocytes and macrophages produce similar amounts of TNF. This supports the idea that macrophages are key in establishing latency via TNF production, while T cell–derived TNF is essential, but not sufficient, for protection against Mtb infection, as shown in experimental data in mice [38].

### Active TB

As discussed in the Methods section, by choosing different sets of parameter values, the mathematical model can simulate active infection. Active TB is characterized by uncontrolled growth of intracellular and extracellular bacteria throughout the simulation (500 days), reaching a total bacterial load of 10^8^ per cm^3^ of granulomatous tissue approximately at day 300 (Figure 2A). Resident macrophage numbers drive cytokine dynamics in the first 300 days. When resident macrophage populations begin to fall (because they all become infected), a switch in bacterial populations occurs: extracellular bacteria are continuously increasing due to MIs bursting while intracellular bacteria reach a saturation level (determined by the level of available macrophages). High bacterial load is coupled to very high levels of IFN-γ (Figure 2D) and TNF (∼1000 pg/ml, see Figure 2E). Total macrophage population increases within the first 200 days, and by one year post-infection most of them are infected (see Figure 2F). T cell numbers (Figure 2B and 2C) are comparable with macrophage numbers during active TB (approximately 10^5^ cells per cm^3^ of granulomatous tissue). IL-12, IL-10 (Figure 2D), and IL-4 levels (Figure 2 E) are also qualitatively and quantitatively similar to those observed in murine and NHP models as well as from limited human studies. For more details, see [31].

### Uncertainty and Sensitivity Analysis

We investigate the importance of specific TNF-dependent mechanisms that allow for infection control via sensitivity and uncertainty analyses (see Methods). We observe how variations in different sets of parameters affect bacterial load. Table 2 illustrates TNF-dependent factors that, when varied, promote either lower bacterial levels (from latency) or increased bacterial load and reactivation of latent TB.

**Table 2:**
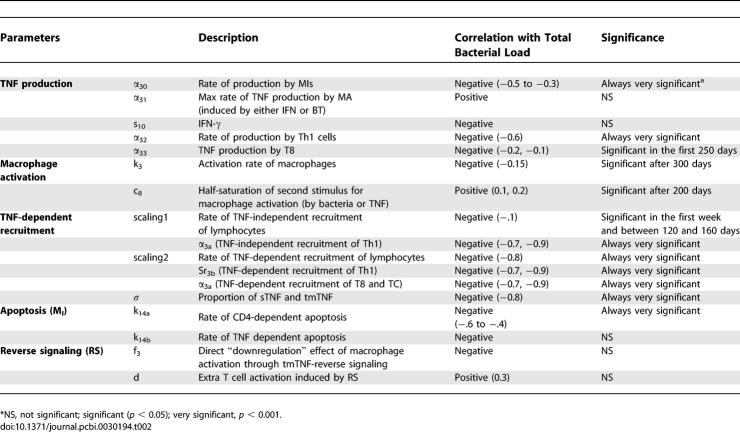
Uncertainty and Sensitivity Analyses of the Model for TNF-Related Parameters

#### TNF production.

Our sensitivity analysis indicates a critical role for TNF production by both MIs (α_30_) and Th1 cells (α_32_) throughout the infection (negative correlation values −0.6 to −0.2, *p* < 0.001, Table 2). TNF production by CD8+ T cells (α_33_) is only significant in the first 250 days post-infection, suggesting that it is important for establishing latency but not maintaining it (see Table S2 for TNF production per cell type during latency).

### Cell Recruitment and Activation

The model predicts that enhanced recruitment of lymphocytes (Th1, T8, and TC) is a desirable strategy toward establishing latency, as suggested by the strong and very significant negative correlation of TNF-dependent recruitment parameters (*Sr_3b_* and *α_3a_*) with bacterial load throughout the course of infection. On the other hand, macrophage activation plays a more important role in maintaining latency: the correlation between macrophage activation rate (*k_3_*) and bacterial load is significant only after latency is achieved (<1 year). TmTNF effects on macrophage (*f_3_*) and lymphocyte activation (*d*) are not significant in either achieving or maintaining latency.

Among all TNF-related mechanisms, the uncertainty and sensitivity analyses indicate that lymphocyte recruitment and macrophage activation are the most influential toward controlling bacterial levels when compared with TNF-induced apoptosis (which is not significant, Table 2). This is consistent with experimental evidence that TNF-induced apoptosis accounts for only 5%–10% killing of MIs [39,40]. Our virtual deletion and depletion experiments are consistent with TNF gene knockouts and TNF neutralization studies (Methods), further supporting that TNF is necessary toward both achieving and maintaining latency.

### A Crucial Role of sTNF in Achieving and Maintaining Latency

**Figure 3:**
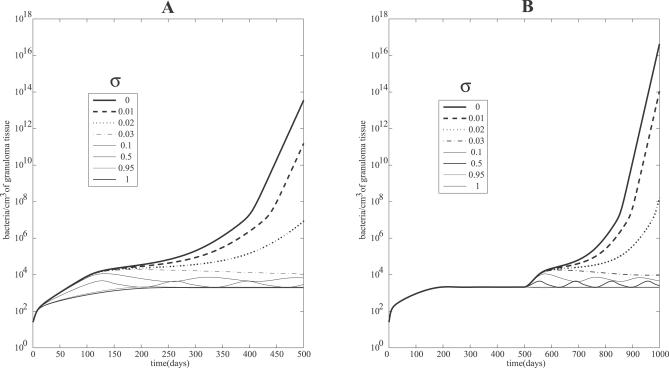
Comparing the Roles of sTNF and tmTNF (A) Mathematical model simulations of total bacterial load corresponding to different proportions *σ* (percent sTNF versus tmTNF); all the other parameters are fixed to parameters yielding a latent state (see Table S6). (B) Simulated depletion of variable levels of sTNF. Until day 500, the system is in latency and *σ* = 0.95. Then at day 500, the depletion of sTNF is performed. Different values of *σ* are shown, where *σ* is the percent cleaved TNF (sTNF). Total bacterial loads are shown corresponding to different percentages of sTNF after day 500.

Little or no data are available to indicate the fraction of TNF (*F_α_*) that is cleaved into sTNF in vivo. We introduce a parameter *σ* to indicate the fraction of TNF cleaved. The remaining (1 − *σ* )*F_α_* represents tmTNF (where sTNF + tmTNF = *σ F_α_* + (1 − *σ* )*F_α_* = *F_α_*. Considering the relative transient expression of tmTNF in vitro [41], we assume that *σ* is approximately 95% (i.e., only 5% TNF is transmembrane) so that the majority of tmTNF is cleaved and released in its bioactive soluble form. We test variations on levels of *σ* (percent sTNF) and report results in two settings in Figure 3. First we explore different percentages of sTNF (*σ*) and look at the effects on bacteria load, and then we deplete different levels of *σ* after latency has been attained.

Figure 3A shows bacterial load for different percentages *σ* cleaved TNF. The system gradually shifts to higher bacterial loads with decreasing amounts of sTNF. This transition arises through oscillations that push the system to active TB when sTNF is almost completely deleted (where sTNF is <3% of total TNF). We obtain a similar dynamic during a depletion experiment where at day 500 (after latency is attained) we deplete varying levels of sTNF from the system (Figure 3B). The system reactivates when almost no sTNF is released. This suggests sTNF is necessary to control active TB and to maintain latency, likely because of its crucial role in lymphocyte and macrophage recruitment, and that tmTNF is not sufficient to maintain latency in humans, as seen in mice [42,43]. Figure S3 numerically shows how the stability of the latency state is dependent on *σ* and partially explains why sTNF is necessary to maintain latency (as shown in Figure 3B).

### Anti-TNF Therapies

We use the mathematical model to simulate three virtual clinical trials (VCT) of anti-TNF treatments (protocols are described in detail in Tables S3 and S4). The first two VCT are designed to explore which factors contribute most to reactivation of latent TB during two types of anti-TNF treatment. The third VCT explores the effects of exposure to Mtb after anti-TNF treatment is initiated.

Two classes of biological inhibitors were tested in the VCT: anti-TNF antibody and TNF receptor fusion (TNFR2Fc). We define each drug as having a specific ability to neutralize TNF at the granuloma site; these data are not currently known (i.e., the drug neutralizing power). We define *TNF bioavailability* as the amount of total TNF available for use in the granuloma during anti-TNF treatment. Since we model TNF concentrations in granulomatous tissues, high bioavailability of TNF during therapy translates into a low neutralizing power of the drug or low penetration of the drug into granulomatous tissue.

As shown above (Figure 1), our simulation of the latent TB state predicts TNF levels at 0.12 pg/mL. This is the same order as those obtained via our granuloma homogenate data of 0.5 pg/ml derived from a nonhuman primate model (see Text S2). When performing deletion and depletion experiments, we determined that only a small percentage of the total latency TNF was required to maintain latency (see Methods). At levels of TNF below that minimum, the system always reactivated. We assume treatment affects TNF bioavailability such that only a percentage of the latency level is bioavailable. We define *reactivation threshold* (RT) as a level (percent of TNF in latency) of bioavailable TNF below which results in reactivation (see Methods). Thus, during anti-TNF treatment we can predict whether the bioavailable TNF is pushed below this threshold, leading to reactivation. If the RT is high, then more bioavailable TNF is required to maintain latency; if RT is low, only very low levels of bioavailable TNF are needed to maintain latency.

### Virtual Clinical Trial 1

A series of VCT were simulated assuming different TNF bioavailability ranges induced by the two different treatments and a natural biological variation of *σ* (percentage of total TNF cleaved and released as sTNF). Considering the transient expression of tmTNF in vitro [41], we assume *σ* varies between 50% and 100%. Table 3 illustrates the results in terms of number of reactivations per 100 latently infected virtual patients.

**Table 3:**
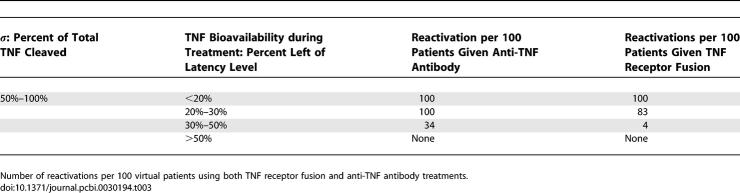
Virtual Clinical Trial 1 (VCT1)

If TNF bioavailability at the granuloma site is <20% of baseline latency level (at the initiation of therapy at 500 days), both treatments induce 100% reactivation. In the range 20%–30%, anti-TNF antibody always causes reactivation, while TNFR2Fc reactivates 83/100 virtual patients. At higher bioavailability ranges (30%–50%), the differential risk of reactivation goes down to 34 or 4 per 100 virtual patients for anti-TNF antibody versus TNFR2Fc, respectively. No reactivation occurs for both treatments if more than 50% of TNF is bioavailable. The prediction that anti-TNF antibody treatment has a stronger impact on reactivation risk than TNFR2Fc in the bioavailability range of 20%–50% suggests that other factors may be playing a role in reactivation in addition to bioavailability. To explore this, we simulated another clinical trial.

### Virtual Clinical Trial 2

In addition to bioavailability, the percentage of total TNF cleaved (*σ*) may also be an important variable. First, we will assume that both molecules equally affect TNF bioavailability so we can explore independently the effects of sTNF versus tmTNF levels. Table 4 shows results for evaluating RT for different *σ* to investigate the role of tmTNF in TB reactivation during anti-TNF therapy. If we assume that *σ* is not very variable in the population and approximately equal to 95% (the baseline value that we used to generate our latency results), and we vary TNF bioavailability in the same range for both treatments (between 20% and 50%), our model predictions show that the differential reactivation risk is 34 per 100 anti-TNF antibody-treated virtual patients versus 28 per 100 TNFR2Fc-treated virtual patients (Table 4). RTs are not significantly different for this first experiment (25.11% versus 24.19%). However, if *σ* is allowed to vary from 50% up to 100%, as in VCT1, the VCT now predicts a differential reactivation risk of 46 per 100 anti-TNF antibody-treated virtual patients versus 30 per 100 TNFR2Fc-treated virtual patients (Table 4). RTs are now significantly different (28.62% versus 25.01%, *p* < 0.001). So, a lower *σ* (more tmTNF and less sTNF) has a negative impact on maintaining latency during anti-TNF antibody treatment.

**Table 4:**
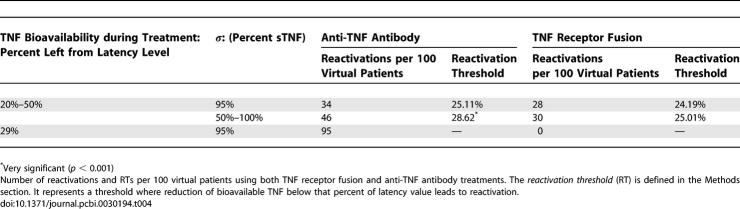
Virtual Clinical Trial 2 (VCT2)

To determine other factors that contribute to reactivation differences between the two therapies, we now fix both TNF bioavailability and *σ*. Since the RTs were not significantly different between the two treatments when *σ* is fixed at 95%, we isolate the effect of TNF bioavailability by fixing it at 28%. We chose this value of bioavailability specifically because it allows us to determine other factors responsible for more reactivation in the anti-TNF antibody therapy when no reactivations occur for the TNFR2Fc therapy (Table 4).

Our sensitivity analysis (Table S5) demonstrates that by varying all 12 parameters in the model that are affected by anti-TNF antibody treatment, only three contribute significantly to the observed reactivation. Cell loss rates of IFN-γ producing CD8+ cell (T8 [31]) and MA induced by anti-TNF antibody therapy positively correlated with total bacterial load (μ_tmTNF-MA_ and μ_tmTNF-T8_).

The cell loss rate of MI negatively correlated with total bacterial load (μ_tmTNF-MI_). Thus, anti-TNF treatment reduces the number of MAs and T8 cells and increases bacterial levels, increasing risk of reactivation. Although anti-TNF antibody also reduces the number of MIs, this is not sufficient to maintain latency. This may explain why a higher percentage of tmTNF has a negative impact on infection containment during anti-TNF antibody treatment: with more tmTNF, more MAs and T8 cells are lost from the granuloma.

Duration of treatment also affects risk of reactivation for both therapies. Table 5 shows simulation results where we varied simultaneously TNF bioavailability (between 20% and 50%) and *σ* (between 50% and 100%), setting different protocols for treatment duration. There is a significant difference in average RTs between a 12-month regimen and 18- or 24-month regimen for both treatments. We tested whether bacterial load at treatment initiation affects reactivation risk. If the total bacterial load is <500 CFU, no reactivation is observed for both treatments. If total bacterial load is 2–3 fold higher than latency level (∼ 3–4e3 CFU), the system always reactivates for both treatments (unpublished data).

**Table 5:**
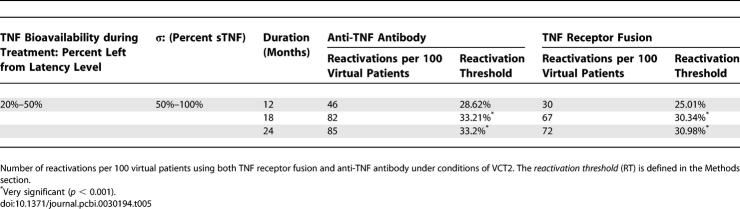
Duration of Treatment study for VCT2

### Virtual Clinical Trial 3

If treatment starts before infection with Mtb occurs, we assume that drug penetration is not relevant because the granuloma has not yet formed. We assume instead that a certain concentration of anti-TNF molecules are present in the lung where granulomas would begin to form in response to infection. Average serum concentrations of anti-TNF molecules are published [44] but no data are available for lung. It takes 300 days to achieve latency with TNF at 0.12 pg/mL (see Figure 1). We capture changes in TNF bioavailability during anti-TNF therapy by dynamically lowering TNF levels by a constant percentage throughout treatment (Figure S1). For level of total TNF 50% lower than baseline latency levels, treatment with either drug leads to disease, following acute infection, with no significant differences between them (Figure S2).

## Discussion

The major findings from this study are that bioavailability of TNF following anti-TNF therapy is the primary factor for causing reactivation of latent infection, that anti-TNF therapy will likely lead to numerous incidents of primary TB if used in areas where exposure is likely, and that sTNF—even at very low levels—is essential for control of infection.

Our model predictions (see Figure 3) recapitulate recent murine studies that tmTNF is sufficient to provide acute but not long-term control of Mtb infection [42,43]. We predict that >2% of total TNF needs to be released in soluble form to control acute infection and maintain latency. Bacterial loads increase as the percentage *σ* of total TNF cleaved is decreased, i.e., allowing more tmTNF in the system.

We use the model to analyze the effects of anti-TNF therapy by simulating anti-TNF antibody and TNFR2Fc. The reported measure unit for a steady state or average concentration of anti-TNF drugs in serum is on the order of μg/ml. Data on soluble TNFRs concentration in serum are on the order of ng/ml [45]. We use and predict concentrations of sTNF within the order of pg/mL based on our granuloma homogenate data (see Text S2). Given these predicted and reported concentrations, both treatments can systemically neutralize most if not all TNF. We can speculate that both TNFR2Fc and antibody will likely neutralize most if not all TNF at the granuloma site, if each penetrates granulomatous tissue equally well. An alternative way to interpret bioavailability is in terms of drug penetration into granulomatous tissues. However, our studies in murine models suggest that anti-TNF antibody penetrates or remains in the granuloma at higher levels than receptor fusion molecules [46]. The pharmacodynamic differences between these two agents include increased dissociation rate of TNF from TNFR2Fc compared with antibody [47]. Whether these differences play a role inside the granuloma is not known. However, one can imagine that increased dissociation in the context of high concentrations of endogenous TNF receptors could lead to better TNF signaling in the granuloma.

The VCT simulations suggest that TNF bioavailability is the main factor leading to reactivation by anti-TNF treatments in latently infected patients. Reactivation always occurs if both drugs penetrate the granuloma equally well (TNF bioavailability less than 20%). High bacterial load at treatment initiation increases the likelihood of reactivation. This suggests that a complete regimen of antibiotic treatment for Mtb infection prior to anti-TNF treatment could reduce the risk of reactivation. If TNF bioavailability is equally affected by the two treatments, differential cell level losses induced by anti-TNF antibody therapy accounts for higher reactivation rates: activated CD8+ T cells and MA loss are not compensated by the apparently beneficial effect of MI loss. We speculate that the intracellular bacteria released after MI death induced by antibody binding to tmTNF (whether dependent on tmTNF reverse signaling or complement cascade) can only facilitate bacterial clearance by the host and does not enhance dissemination. Further, our results show that the longer patients are exposed to anti-TNF drugs through longer duration treatment protocols, the risk of reactivation increases. If infection with Mtb occurs after treatment is initiated, chances of developing active infection are very high if we assume reasonable levels of drug penetration into lungs (TNF bioavailability <50%). This is particularly important if anti-TNF treatments are implemented in regions of the world where infection risk is elevated. Bacteria grow uncontrolled when both sTNF and tmTNF are depleted (anti-TNF antibody therapy).

These data suggest that tmTNF plays a key role in controlling active infection, where tmTNF preserves a subset of the beneficial mechanisms of TNF while lacking detrimental effects. Our predictions and recent experimental data [42] support the hypothesis that selective targeting of sTNF may offer several advantages over complete blockade of TNF in the treatment of chronic inflammatory diseases.

Current studies in both murine and NHP animal models by our group are now following up on these predictions. Our recent data from a mouse model showed that treatment with anti-TNF Ab in the chronic phase rapidly resulted in fulminant TB, while treatment with an etanercept-like molecule (receptor fusion) allowed mice to maintain *control* of the infection [46]. In contrast, following treatment with either antibody or receptor fusion during initial infection caused mice to succumb rapidly to the infection. This clearly demonstrated, in a mouse model, that there are differences between the two TNF neutralizing drugs depending on the phase of infection [46]. Our studies in NHP model are ongoing. Finally, this work focuses on reactivation based on bacteria harbored in the lungs. But data exist suggesting that bacteria do not reside only in granulomas, but in other places in the body during latency. For example, recent data by Neyrolles et al. [48] support the presence of nonreplicating bacteria in adipose tissue during latent TB infection. In the future we can consider the role of reseeding of the lungs from other body sites as a possible feature in reactivation.

## Methods

To better understand underlying dynamics of TNF production and function, we build on our mathematical model of Mtb in humans using 16 nonlinear ordinary differential equations. The updated model tracks three macrophage populations (resting, activated, and infected) and multiple T cell (Th0, Th1, Th2, and CD8+ T cell subsets) populations [31]. The model also includes five cytokine concentrations (IFN-γ, IL-12, total TNF, IL-10, and IL-4) and two bacterial (intracellular and extracellular) populations with numbers representing temporal dynamics of these populations in the lung (our modeling environment). The biological assumptions and the equations of the updated human Mtb model are outlined and described in the following section.

### Model equations.

**Figure 4:**
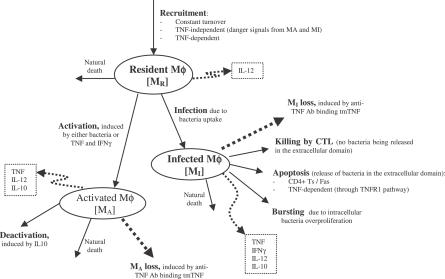
Macrophage Dynamics Descriptive diagram of macrophage dynamics implemented in the mathematical model in Equations 1–3.

**Figure 5:**
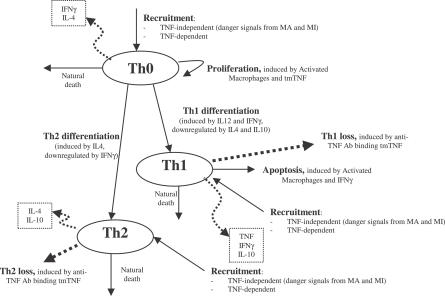
CD4+ T Cell Dynamics Descriptive diagram of lymphocyte dynamics implemented in the mathematical model in Equations 4–6.

**Figure 6:**
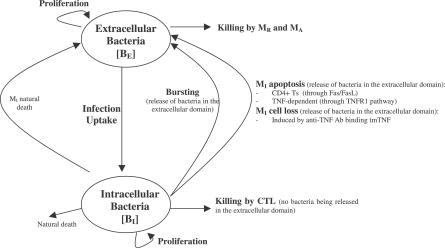
Bacteria Dynamics Descriptive diagram of bacteria dynamics implemented in the mathematical model in Equations 15–16.

The nonlinear ODE model is based on [31] and simulates interactions between two bacterial subpopulations, eight cell populations, and five cytokines. The new terms related to TNF dynamics are represented in bold and are described in the Methods section. Submodel diagrams are illustrated in Figures 4–6. They show macrophage, lymphocyte, and bacteria dynamics, capturing the terms represented for each of our equations. Cytokine production dynamics are superimposed on each diagram.

### Macrophage dynamics.

The equations describing dynamics for the macrophage subpopulations are given by:

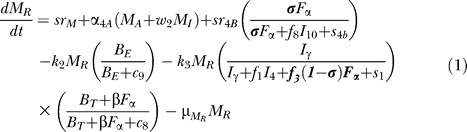


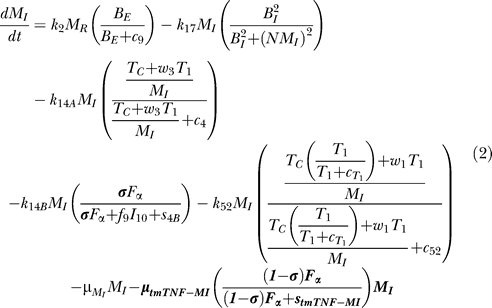


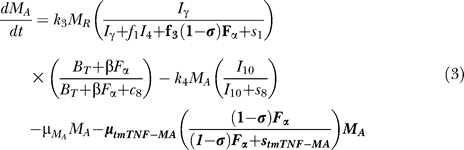

Figure 4 shows a descriptive diagram of macrophage dynamics, with the terms captured for each of our equations.

Rate of change of resting macrophages (Equation 1) includes a source term (sr_M_) and a natural death term (-μ_M_RM___R_). In the course of infection, additional resting macrophages are recruited in a TNF-dependent fashion at a rate Sr_4B_, and this process is downregulated by IL-10. We also account for TNF-independent recruitment mechanisms (for both macrophages and lymphocytes) with a term that indirectly represent chemokines secreted primarily by MAs and MIs (*α*(M_A_ + w M_I_), 0 < w < 1): the magnitude of recruitment (α) varies from macrophages to lymphocytes. Resting macrophages at the site of infection can become chronically infected at a maximum rate k_2_ (dependent on the extracellular bacterial load) and activated at rate k_3_ (dependent on two signals from IFN-γ and either bacteria or TNF). Note that due to differences in measurement units, TNF is scaled by a factor β. IFN-γ induction is downregulated by IL-4.

MIs (Equation 2) can be cleared by one of several different mechanisms. Given an average maximal intracellular bacterial carrying capacity of N, we assume that one-half of the MIs burst when the intracellular bacterial load reaches NM_I_. This mechanism has a maximal rate k_17_, and is described by a Hill process. Immune responses also contribute to MI killing by several mechanisms. Both CD8+ and CD4+ T cells can use the Fas-FasL apoptotic pathway to induce apoptosis in these cells at a maximum rate k_14a_. The half-saturation constant c_4_ describes the effector-target ratio (T_t_:M_I_) at which this process is half maximal. TNF can also induce apoptosis by binding to the TNFR1 receptor. This process is downregulated by IL-10 and occurs at a rate k_14b_. Finally, CTL killing by CD8+ and CD4+ T cells happens at a rate of k_52_. Specifically, CD4+ T cells have a limited contribution and this is accounted for by scaling the CD4+ T cell numbers (0 < w_1_ <1). CD8+ T cell numbers are scaled by a Michaelis-Menten term accounting for the indirect dependence on CD4+ T cells for their killing capability. MAs are generated from the term in Equation 1 and undergo natural death at a rate proportional to their number (-μ_M_AM___A_). MAs can be deactivated by IL-10 at a rate k_4._

### T cell dynamics.



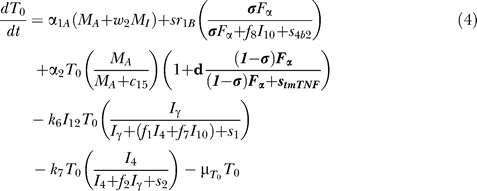


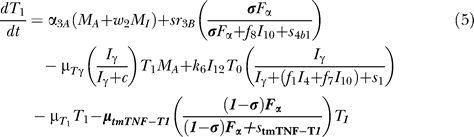


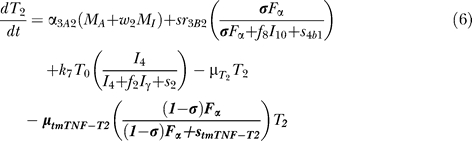


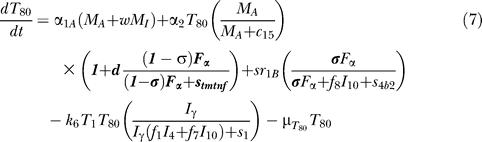


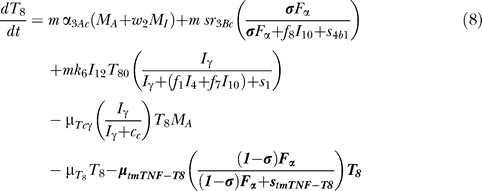


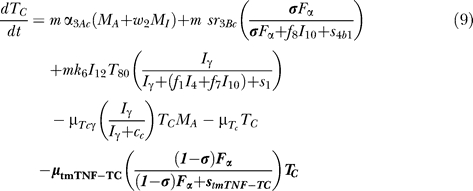



Figure 5 shows a descriptive diagram of CD4+ lymphocyte dynamics, with the terms captured for each of our equations. A similar diagram can capture the dynamics of CD8+ lymphocytes (Equations 7–9).

Similar to resting macrophages, recruitment of T cells occurs in both a TNF-independent and a TNF-dependent manner. The terms are similar, using different rates for the different T cell subsets (α_1A_, Sr_1B_ for Th0 and T80 cells; α_3A_, Sr_3B_ for Th1 and Th2 cells; α_3Ac_, Sr_3Bc_ for CD8+ T cells, respectively). We assume that CD4+ T cells can arrive at the site of infection either as Th0 (majority), or a small fraction may arrive already differentiated intoTh1 or Th2 cells (see Wigginton et al. [28] for a complete discussion).

Upon arriving at the site of infection, Th0 cells (Equation 4) can proliferate further in response to signals released by MAs at a rate α_2_. Th0 cells can also differentiate into Th1 (Equation 5) and Th2 (Equation 6) cells. Th1 differentiation is controlled by IL-12 and IFN-γ and opposed by IL-4 and IL-10. Th2 differentiation is induced by IL-4 and inhibited by IFN-γ. Th0 cells undergo natural death at a rate (-μ_T0_T_0_). Th1 cells can be killed due to IFN-γ induced apoptosis in the presence of MAs at a rate μ_Tγ_. Both Th1 and Th2 cells die naturally at rates μ_T1_ and μ_T2_, respectively. As is the case for CD4+ T cells, we assume that CD8+ T cells can arrive at the site of infection as T80 (majority) (Equation 7), or a small fraction may arrive already differentiated into effector cells of either T8 (Equation 8) or TC (Equation 9) type. T80 cells are activated due to interaction with Th1 cells and cytokines and have a natural half-life.

CD8+ T cells also undergo IFN-γ induced apoptosis at a peak rate μ_Tcγ_, and die at a rate μ_Tc_. Since the T8s (Equation 8) and Tcs (Equation 9) are functional subsets of the CD8+ T cell population (see Introduction), the equations are identical for both. We introduce a parameter m that accounts for possible overlap between T8 and TC subsets. This assumption is studied further in the CD8+ T cell kinetics section of Sud et al. [31].

### Cytokine Dynamics.



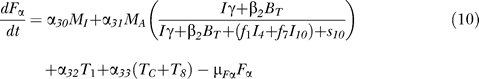


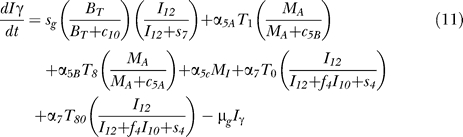


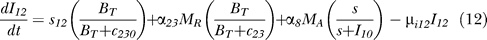


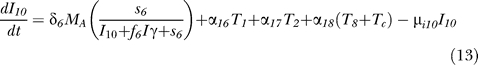


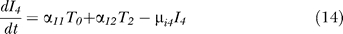



TNF (Equation 10) is produced primarily by MIs at a rate α_30_. MAs make TNF at a rate α_31_ in response to IFN-γ or bacteria and this process is inhibited by IL-10 and IL-4. Other sources of TNF are Th1 cells (rate α_32_) and CD8+ T cells (rate α_33_) in response to antigen, and TNF has a given half-life.

Th0, Th1, and CD8+ T cells produce IFN-γ (Equation 11) in response to antigen presentation by MAs at rates α_5A_ and α_5B_, respectively. Production by Th0 and T80 cells is further enhanced by IL-12, and inhibited by IL-10. Other sources of IFN-γ, such as NK cells, are also believed to play a role in TB infection. Since these are not accounted for in the model, we include an extra source term (s_g_) dependent on extent of infection and IL-12 level.

Resting macrophages produce IL-12 (Equation 12) in response to infection at a rate α_23_. MAs also produce IL-12, and this process is downregulated by IL-10. Dendritic cells are the primary source of IL-12 upon Mtb infection and are accounted for by an infection-dependent source term, s_12_. Finally, there is a natural half-life for IL-12.

IL-10 (Equation 13) is produced mostly by MAs, and this process is opposed by IFN-γ and IL-10 itself at rate δ_6_. Other sources such as Th1 cells, Th2 cells, and CD8+ T cells produce IL-10 at rates α_16_, α_17_, and α_18_, respectively. IL-4 is produced by Th0, and Th2 cells produce (Equation 14) at rates α_11_ and α_12_, respectively. IL-4 has a given half-life of μ_i4_.

### Bacterial dynamics.

Figure 6 shows a descriptive diagram of bacteria dynamics, with the terms captured for each of our equations.

Intracellular bacteria (Equation 15) grow at a maximal rate α_19_ with logistic Hill kinetics accounting for a maximal carrying capacity of a macrophage. Extracellular bacteria (Equation 16) become intracellular when a macrophage becomes chronically infected at an assumed threshold of N/2 bacteria, and hence this represents a gain term for the intracellular bacteria. Bursting of macrophages (k_17_) adds to the extracellular subpopulation. To account for loss of intracellular bacteria due to various killing mechanisms, we assume each killed MI to hold an “average” number of bacteria, given by N_AVG_ (<=N). The corresponding gain in extracellular bacteria depends on the mechanism of killing: while Fas-FasL–induced apoptosis (k_14a_) releases all intracellular bacteria, TNF-induced apoptosis (k_14b_) eliminates approximately 50% of the bacteria within the macrophage, and this is shown by the N_frac_a__ multiplier in the BE (extracellular bacteria) equation (Equation 16). CTL activity (k_52_) kills virtually all the intracellular bacteria, and does not add on to the BE (extracellular bacteria) population. Lastly, we assume that natural death of MIs also releases all intracellular bacteria, and this is modeled as a constant turnover of the bacteria (μ_I_B_I_) from intracellular to extracellular. Extracellular bacteria grow at a maximum rate α_20_. They are taken up and killed by activated and resting macrophages at rates k_15_ and k_18_, respectively.

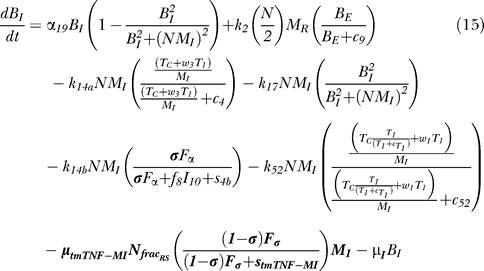


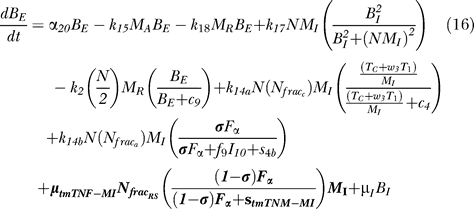



### Representing soluble and transmembrane TNF in the model.

sTNF is produced predominantly by cells of the macrophage lineage upon infection or exposure to bacteria or bacterial products [49]. Other cell types producing sTNF include T cells and NK cells [50]. Stimuli for sTNF production also include chemokines or cytokines (CCL3, IL-1) and also cellular stress responses such as hypoxia, oxygen radicals, and temperature shock.

Our previously published models of Mtb infection simulated cell recruitment as a function of MAs and MIs, the main producers of chemokine and sTNF. In our most recent model [31], TNF was explicitly modeled in its soluble form. A TNF-dependent recruitment term for both macrophages and T cells was included, while maintaining previous terms to account for additional recruitment not dependent on TNF [6]. Here we modify the existing model to include tmTNF and its effects (see Table 1). TNF (labeled as F_α_) represents the dynamics of total sTNF and tmTNF in the system. Using the model, we investigate how different percentages of total TNF cleaved (i.e., sTNF) affect infection progression. We updated the model equations to address tmTNF effects on cell activation and apoptosis, based on Table 1. A direct effect of tmTNF in T cell activation (both through TNF receptors and tmTNF reverse signaling) is included in the equation for T0 and T80 (bold term):

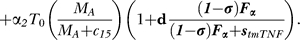



The strength of tmTNF effect on T cell activation through TNFR1 and TNFR2 is represented in the model by the coefficient *d*.

We also add new terms representing apoptosis or cell loss induced by anti-TNF antibody binding to tmTNF on macrophages [19,20] and T cells [51]. The cell-loss terms for both MIs and MAs, as well as lymphocytes, are implemented as follows

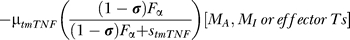



These terms are present in the mathematical model only during anti-TNF antibody treatment simulations. The fraction of intracellular bacteria released in the extracellular domain due to tmTNF-induced apoptosis of MIs is likely very small [25]. A new term describes it by multiplying the number of MIs that die by a fraction of intracellular bacteria being released.

Under pathological conditions (chronic inflammatory states), the presence of anti-TNF antibodies (and not TNF receptor fusion molecules) and subsequent binding to tmTNF can induce activation of the complement cascade (due to high concentration of Abs) [52] and apoptosis induced by reverse signaling through tmTNF binding [19]. Activation of complement cascade is supported by data on Crohn's disease, and it might not be a mechanism shared among all the TNF-related pathologies (such as rheumatoid arthritis and ankylosing spondylitis). The likely consequence of triggering the complement cascade is the release of intracellular bacteria, while apoptosis kills most of the intracellular bacteria [53]. A direct “downregulation” effect of macrophage activation through tmTNF reverse signaling (macrophage anergy or LPS resistance) is included in the macrophage activation term (k_3_) as follows (bold term):

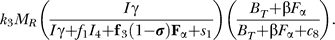



We do not directly include LT_α_ in the model, but we indirectly account for LT_α_-dependent recruitment of macrophages and lymphocytes during anti-TNF therapy (namely TNF receptor fusion protein), since TNF receptor fusion protein binds LT_α_ while anti-TNF antibody does not (see Table S3). We differentiate the two treatments by downregulating all TNF-independent recruitment terms during receptor fusion treatment simulations

### Computer simulations.

Once we derive the model and estimate parameters, we solve the system of 16 nonlinear ordinary differential equations to obtain temporal dynamics for each variable. To this end, we used Matlab version 7.1.0.183 (R14) Service Pack 3 (The Mathworks) platform and its numerical methods together with a computer code using a different solver written by our group.

As discussed previously [28,29,31], we chose total bacterial load as a marker of disease, where bacterial levels can distinguish between two different scenarios: latent infection (steady state, low stable bacterial levels) and active disease (unchecked bacterial growth).

### Parameter estimation.

Before simulations can be performed, parameters must be estimated from literature sources or by mathematical means. Values for most model parameters are estimated from published experimental data or data generated from our group. Data from human studies and Mtb experiments are favored over mice and other mycobacterial species, respectively. Where no appropriate data is available for a given parameter, we conduct uncertainty analysis to obtain a range within orders of magnitude. A detailed description of techniques used to evaluate model parameters, as well as a listing of parameters already estimated can be found in work previously published by our group [31]. All parameters newly estimated for the purpose of this work are listed in Table S6, together with parameters previously estimated. All parameters have been estimated using approaches similar to those described in Wigginton et al. [28].

Parameter values represent mechanisms in the host–pathogen system, and these were estimated from many different experimental sources. There is great variation that likely exists among them. In previous work [31], we explored wide ranges on these parameter values to determine how the system changes when values change. A group of parameters were identified as being key determinants between the host–pathogen system achieving latency or going to active disease (see Table I and II in [31]). These different infection states are obtained by varying parameter values, as discussed in the next section. The set of parameters that we used to generate the simulation of latency (Figure 1) is given in Table S6. Here, we vary only a small subset of these parameters to obtain an active disease simulation (see legend of Figure 2 for details).

### Uncertainty and sensitivity analysis.

There is an intrinsic biological and experimental variability in rates measured from in vivo or in vitro studies. Further, some interactions in the Mtb–host system are not currently measurable, particularly at the level of the lung granuloma. This complicates accurate estimation of model parameters (baseline values are unknown).

We quantify the importance of each TNF-related mechanism involved directly and indirectly in the infection dynamics using both uncertainty and sensitivity analyses as described previously [31]. The statistical techniques of latin hypercube sampling (LHS) and partial rank correlation (PRC) [54–57] when combined guide our understanding as to how and to what extent variability in parameter values affects infection outcomes. We employ the LHS method to control effects of uncertainties in our parameter estimation by varying all the TNF-related parameters simultaneously. LHS allows for simultaneous random, evenly distributed sampling of each parameter within a defined range (stratified Monte Carlo technique). The sampling is done by varying each parameter over a wide range (up to a factor of 1,000 above and below reported literature data or mathematical estimates) and performing a large number of computer simulations (*n* is significantly large). The stochastic approach allows for a global sensitivity study as compared with a deterministic analysis that gives local results based on the sensitivity equations. One major drawback of the deterministic approach is that the results are based on baseline values (often unknown) for the parameters involved in the sensitivity equations. The LHS approach does not suffer from this limitation.

The PRC method allows us to correlate the variability observed using the LHS method and to determine which parameters are responsible for the variation in outcomes. PRC coefficients (PRCCs) are between −1 and 1 and have a standard *p*-value that indicates significance. A negative PRC coefficient indicates that a decrease in the value of that parameter results in an increase of the bacterial load. A positive PRC coefficient indicates that a decrease in the value of that parameter results in a decrease of the bacterial load. PRC coefficients also evaluate temporal changes in the significance of these parameters as they relate to bacterial load at different times during the infection. Statistical significance of these correlations is assessed by a generalized *t*-test (see the “Statistical analysis” section). For example, the rate of TNF production by Th1 cells (α_32_) is always very significant and negative (see Table 2): if we lower TNF production by Th1 cells, bacterial load increases.

### Virtual deletion and depletion.

As a way to validate the mathematical model, we recapitulate experimental approaches such as TNF gene knockouts and TNF neutralization studies. These can be simulated with our mathematical model as virtual deletion and depletion simulations, respectively. Virtual deletions remove an element from the system at day zero while virtual depletions mimic experimental conditions where an element can be depleted or neutralized via antibody treatment at any time during the infection. We can selectively delete or deplete sTNF or transmembrane TNF (tmTNF) by varying the parameter *σ* prior to infection (Figure 3A) or after latency is achieved (i.e., at day 500 post-infection, Figure 3B). Setting *σ* to zero mimics sTNF deletion/depletion, while tmTNF deletion/depletion is obtained by setting *σ* to 1. We restrict our results to virtual TNF deletion/depletion studies to investigate the role of TNF during active and latent TB. Previous deletion and depletion experiments were performed for all of the relevant cells and cytokines in the mathematical model (see [31] for details).

**Figure 7:**
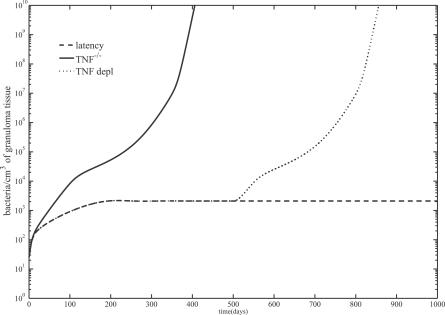
Simulations of Total TNF Deletion and Depletion Mathematical model simulations of bacterial loads during TNF deletion (TNF^−/-^) and depletion (TNF depl). The *y*-axis represents total bacterial load. Latency is our wild-type control (see Figure 1). Note, *σ* = 0.95 for these simulations.

When all the TNF is deleted from the system on the same day that infection is initiated, the system goes to active disease (see Figure 7, TNF^-/-^). This occurs with low-level cellularity, i.e., macrophage numbers are almost an order of magnitude lower (mainly infected and activated) than when disease occurs in the wild-type scenario (see Figure 2). This is consistent with studies that report diffuse infection, where disease is spread throughout a large lung area, resulting in an overall lower cellular density and widespread tissue damage [2–4,11]. Upon total TNF depletion (performed at day 500 post-infection), the system progresses to a disease state in fewer than 100 days (see Figure 7, TNF depl). Depletion reduces the total number of macrophages to 25% (unpublished data), consistent with recent studies [6]. T cells are depleted upon TNF removal mainly because they turn over and are not quickly replenished: they then recover due to compensatory recruitment by other TNF-independent mechanisms in response to high bacterial levels. Thus, the depletion simulations suggest that although TNF is present at extremely low levels during latency (∼0.12 pg/mL of granuloma homogenate, see Figure 1), this low level is necessary and sufficient for control and maintenance of infection. This finding is further confirmed later in the anti-TNF treatment simulations. Our results also indicate that control of infection is a dynamic, TNF-dependent process involving continual cell turnover, an outcome that is consistently observed across experimental studies [8].

### Simulated TNF blockade in tuberculosis infection: Anti-TNF antibody versus TNF receptor fusion.

The US Federal Drug Administration monitors the safety of TNF inhibitors through its Adverse Event Reporting System (AERS), a surveillance system to which drug manufacturers are required to submit reports of adverse events and to which health care professionals and consumers voluntarily send adverse event reports. Wallis et al. [27] published a systematic study of granulomatous infections associated with infliximab and etanercept contained in AERS, using reports from 1998 (when the two drugs were approved) through the third quarter of 2002. TB is the most frequent disease, reported in ∼144 per 100,000 patients (infliximab-treated patients) and in ∼35 per 100,000 patients (etanercept-treated patients). Although the clustering of adverse events reported shortly after initiation of infliximab treatment is consistent with reactivation of latent infection, the number of infected individuals with latent TB is not reported in the AERS database for both treatments. There is a possibility that some TB cases result from infection after therapy is initiated. Anti-TNF antibody treatment (such as with infliximab) targets both sTNF and tmTNF. We also account for additional cell loss due to tmTNF engagement by the drug. TNF receptor fusion treatment (such as with etanercept) targets sTNF and LTα_3_. We capture the action of these two TNF neutralizing drugs by including an additional loss term in the TNF equation. This term accounts for concentration-dependent loss of TNF as a function of a drug's half-life, dissociation rate, bioavailability, and treatment regiment. Table S3 shows data for pharmacokinetics (PK), pharmacodynamics (PD), and treatment protocols (doses and administration) for both drugs. Since our modeling approach describes average dynamics within a granulomatous tissue sample in the lung (see the section “Measure units and modeling space” for details), we define percentages of neutralized sTNF and/or tmTNF (*bioav*) that capture the overall neutralizing power of either receptor fusion or antibody in granulomatous tissue.

### Anti-TNF antibody.

Infliximab is a human–mouse chimeric monoclonal TNF antibody that binds potently and essentially irreversibly to monomeric and trimeric TNF, both soluble and membrane-bound, but does not bind to soluble LTα_3_ [47]. Further, due to the nature of its interaction with tmTNF, infliximab induces apoptosis in TNF-producing cells (including MIs and MAs, CD4+ and CD8+ T cells) via a caspase-dependent pathway [52]. Binding of infliximab to one subunit of trimeric TNF leaves additional subunits free to bind other anti-TNF antibodies, raising the possibility of formation of immune complexes, under certain conditions (i.e., high TNF levels).

### TNF receptor fusion.

Another anti-TNF drug, etanercept, is a TNF receptor p75-IgG fusion protein rather than an antibody. It binds selectively to human trimeric sTNF and LTα_3_, with a 4-fold lower affinity for tmTNF with respect to infliximab [47]. As a result, the effect of a receptor fusion drug is depletive of sTNF, but is less potent in binding tmTNF; thus, little or no apoptosis has been observed in clinical data with this drug. The frequency of reactivation of TB in etanercept-administered patients appears to be lower than for infliximab [27], although head-to-head comparison of the two drugs in human studies have not been performed. While it is possible that differences in apoptotic activity of infliximab and etanercept yield these contrasting results, it is more likely that apoptosis coupled with concentration and timing of TNF neutralization leads to the different outcomes between the two drugs.

Receptor fusion has a fast dissociation rate: it sheds 50% of sTNF and 90% of tmTNF in only 10 minutes, but can bind TNF again immediately [47]. Thus it is possible that the TNF-neutralizing effect is intermittent: molecules of sTNF and LTα_3_ are engaged for very short time intervals, allowing for a redistribution of TNF throughout the system. However, if receptor fusion concentration is relatively high, those released molecules will quickly be reassociated with the receptor fusion and are therefore not free in the system. In contrast, under situations where TNF is released from the receptor fusion molecule and there are high numbers of cell associated TNF receptors present (such as in a granuloma) and possibly a lower level of receptor fusion (due to poor penetration), TNF might bind to the cell-associated TNFR1 or TNFR2 instead of back onto the receptor fusion. This contributes even more to lowering levels of bioavailable TNF in granulomas during receptor fusion treatment.

We indirectly test LTα-neutralization in the model by lowering all the TNF-independent recruitment parameters using the bioavailability coefficient (*bioav*): we assume that LTα-neutralization is of the same magnitude as sTNF neutralization.

### Virtual clinical trials.

We perform three VCTs to investigate what factors contribute most to reactivation during anti-TNF treatments if patients are latently infected or if exposure/infection occurs after anti-TNF treatment is initiated.

Several factors and mechanisms hypothesized to be involved in TB reactivation by anti-TNF drugs can be tested. These include the differential power of the drugs to neutralize TNF bioavailability [47], differential inhibition of TNF signaling events (TNFR1/TNFR2 protein ratio expressed on cell surfaces can serve as a possible path for a cell to direct the consequences of TNF signaling [58]), and differential induction of target cell death induced by anti-TNF antibody binding to tmTNF [19,52]. Using the model, we can directly test the power of the drugs to neutralize TNF by varying the bioavailability parameter *bioav*. We can simultaneously explore differential cell level losses by varying tmTNF-related parameters. Since we do not model TNF receptors, we currently cannot address the other hypothesis (TNFR1/R2 protein ratio). Finally, we investigate both the role of different bacterial loads during latency at treatment initiation and the duration of therapy as additional factors affecting risk of reactivation. To test whether bacterial levels play a role in reactivation rates, we vary two parameters that yield latency scenarios with higher bacterial levels before initiation of treatment (i.e., maximal rates of macrophage activation and CTL killing). The duration of both anti-TNF antibody and receptor fusion treatments varies between 12 and 24 months. We classify a virtual patient as undergoing TB reactivation when the bacterial load grows larger than 10^5^ (latency level) during or after the end of the treatment. See Table S4 for details on the VCT settings.

### Reactivation threshold.

We define a *reactivation threshold* (RT) as a threshold where reduction of bioavailable TNF below this threshold level leads to reactivation. This value is expressed as a percentage of the TNF concentrations defined from the latency value.

Each VCT comprises 100 simulations, where TNF bioavailability is varied in a specified interval. Each run is classified based on the bacterial load level, and reactivation is defined when bacterial loads grows uncontrolled. We define the reactivation subset of the 100 runs as the collection of all the reactivation cases with their bioavailabilities (from the uncertainty analysis). We obtain our RT as the average TNF bioavailability calculated on the reactivation subset. We statistically compare RTs between different trials by a standard *t*-test (see the “Statistical analysis” section).

### Measure units and modeling space.

Contradictory data exist regarding levels of sTNF and sTNF receptors in lung epithelial lining fluid obtained by bronchoalveolar lavage [45,59] in active pulmonary TB and healthy subjects. Very limited data are available on concentration profiles of TNF antagonists outside serum. We can assume that the concentration of the drug in the plasma (or serum) is proportional to the average drug concentration in its whole volume of distribution. The lung is highly vascularized, so average concentrations in plasma could be reasonable proxies for the average concentration of the drugs in the lung. This could be accomplished mathematically by finding a physiological value that translates blood to lung (i.e., volume to space) to account for bioavailability. However, this may not adequately represent diffusion of drug from blood vessels into consolidated granuloma tissue/caseum.

Average steady-state concentrations of anti-TNF antibodies [60] and TNF receptor fusion molecules [61] in serum are a function of the protocol (dose and administration) and type of pathology (see Table S3). TNF receptor fusion ranges from approximately 1 μg/ml up to 6 μg/ml. Anti-TNF antibody ranges from 8 μg/ml to 60 μg/ml (see [44] for details). The modeling space of our most recent model [31] is the whole human lung. Here we adapted that model to represent cellular and bacterial dynamics as number of cells or bacteria per cm^3^ of granulomatous tissue and we describe cytokine concentrations in pg/mL of granuloma homogenate.

### Statistical analysis.

We perform PRC (partial Spearman correlation on rank-transformed data) and *t*-test (one-tail, two-sample unequal variance) with Matlab. See the Uncertainty and Sensitivity Analyses section for more details.

## Supporting Information

Figure S1 Levels of TNF during Virtual Clinical Trial 3 during Both Anti-TNF Treatments (152 KB PDF)

Figure S2 Total Bacterial Loads during VCT3 for TNF Receptor Fusion (A) and Anti-TNF Antibody (B) Treatments (130 KB PDF)

Figure S3Plot of the Gradient versus *σ* (Percentage of Soluble versus Transmembrane TNF)The *x*-axis represents the 16 variables of the ODE system (1–16). The *y*-axis represents the 16 variables of the ODE system (1)-(16). The *y*-axis represents 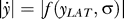 on a log scale.
(54 KB PDF)

Table S1 Summary of Effects of TNF and TNFR1 Blocking during Experimental M. tuberuclosis Infection in Mice (27 KB DOC)

Table S2 Model Simulations of Total TNF Production per Macrophages and T Cell Sources for Different Times (20 KB DOC)

Table S3 Anti-TNF Antibody and TNF Receptor Fusion, Pharmacokinetics, Pharmacodynamics, and Treatment Protocols (21 KB DOC)

Table S4 Summary of Virtual Clinical Trial Protocols and Simulation Conditions (22 KB DOC)

Table S5 Sensitivity Analysis for Anti-TNF Antibody Treatment Simulations (21 KB DOC)

Table S6 Parameter Table New Parameter Estimates in addition to those estimated previously [28,31] (shown in parentheses are the values used to generate a latent state, see Figure 1).
(101 KB DOC)

Text S1 TNF Biology (40 KB PDF)

Text S2 Granuloma Homogenate and Symbolic Analysis (40 KB PDF)

## Abbreviations

AERS: Adverse Event Reporting System
LHS: latin hypercube sampling
MA: activated macrophage
MI: infected macrophage
Mtb: *Mycobacterium tuberculosis;*
PRC: partial rank correlation
sTNF: soluble tumor necrosis factor
TB: tuberculosis
tmTNF: transmembrane TNF
TNF: tumor necrosis factor, VCT, virtual clinical trial
