# Supplementary figures and images for: Differences in Reactivation of Tuberculosis Induced from Anti-TNF Treatments Are Based on Bioavailability in Granulomatous Tissue
Source: PLoS Comput Biol. 2007 Oct 19;3(10):e194. doi: 10.1371/journal.pcbi.0030194 (PMC2041971; doi:10.1371/journal.pcbi.0030194)

## Total TNF concentrations

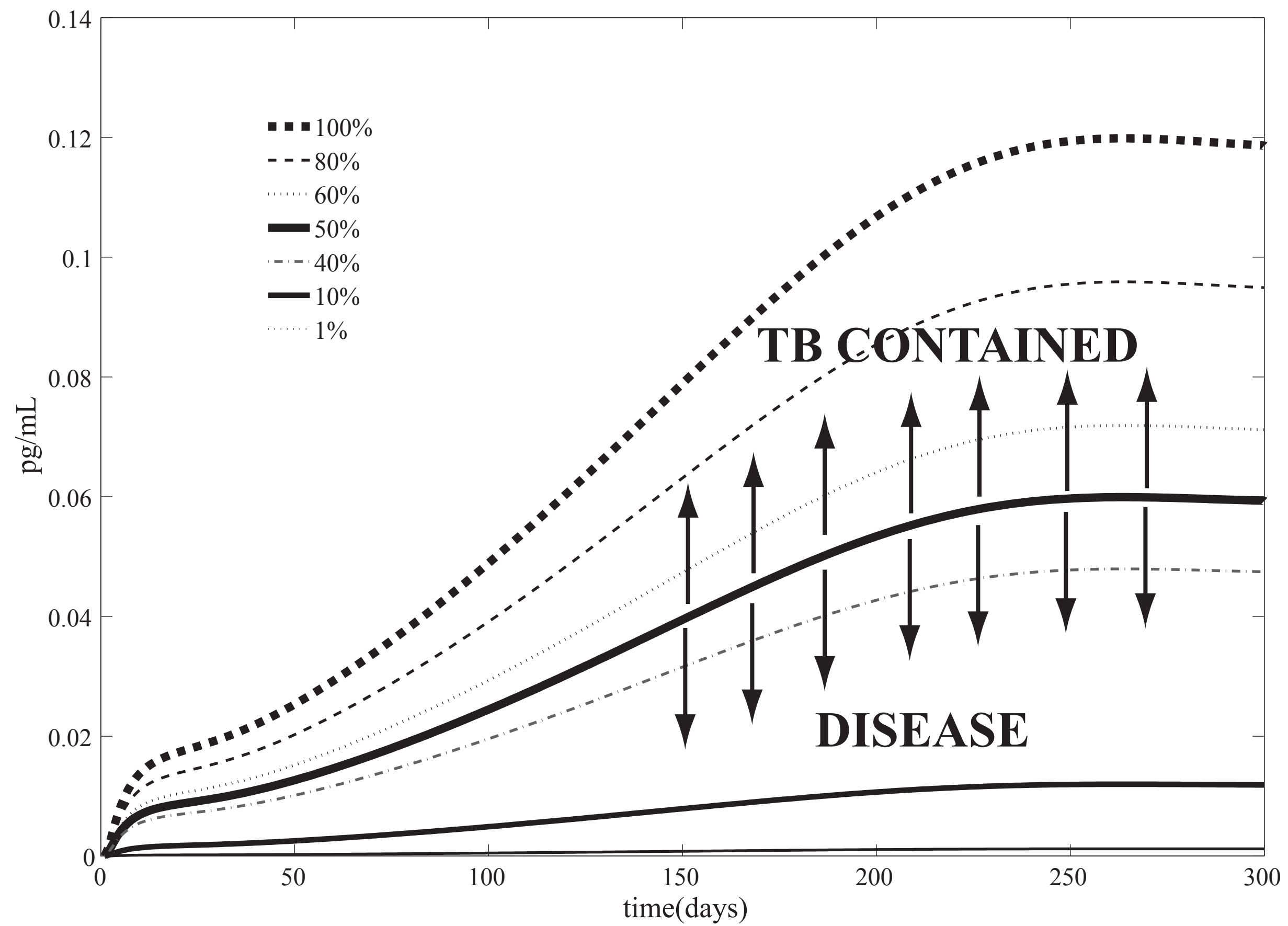

Supplement: Figure S1 — (152 KB PDF) [file pcbi.0030194.sg001.pdf]

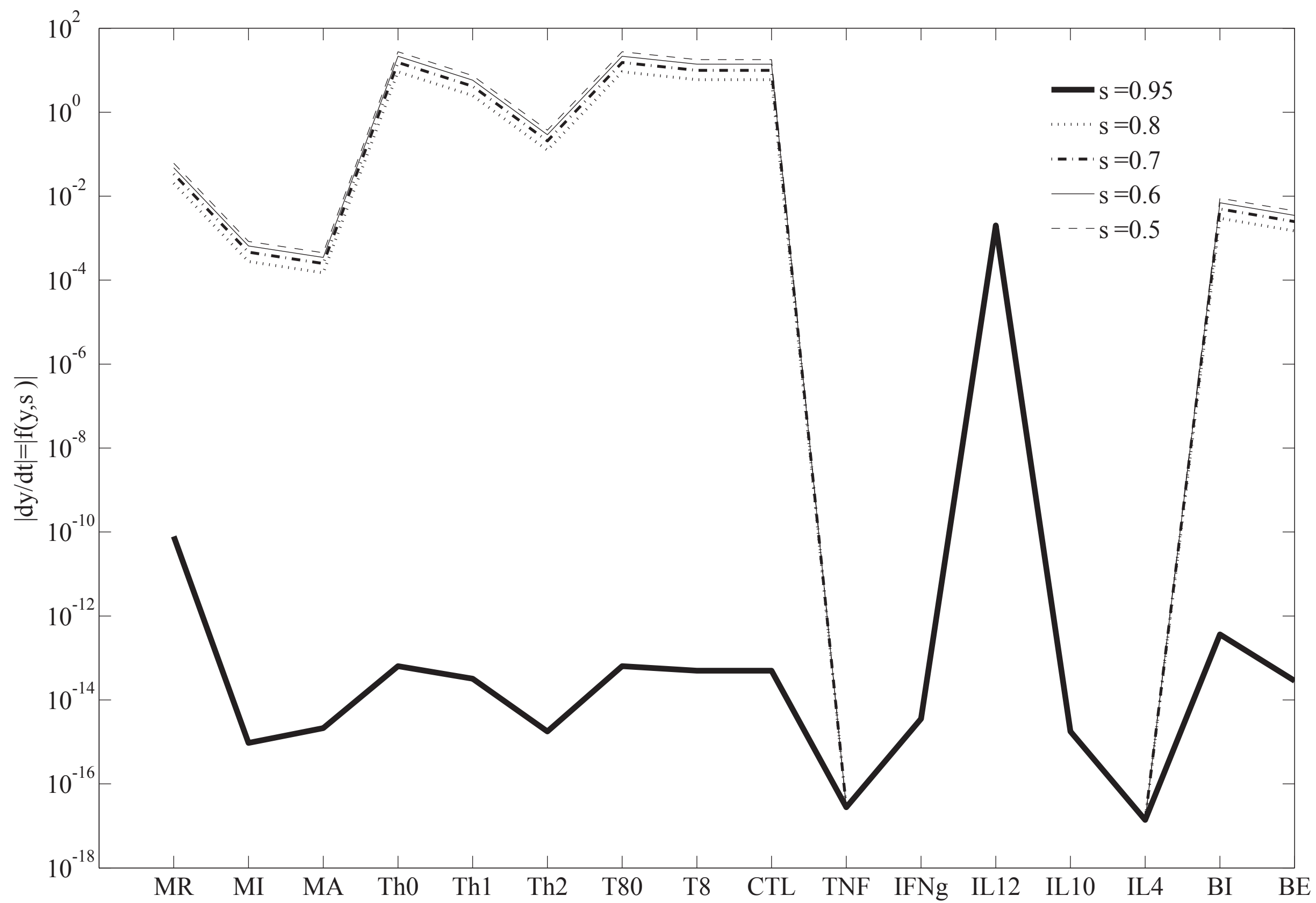

Supplement: Figure S3 — The x-axis represents the 16 variables of the ODE system (1–16). The y-axis represents the 16 variables of the ODE system (1)-(16). The y-axis represents on a log scale. (54 KB PDF) [file pcbi.0030194.sg003.pdf]
