# Supplementary material for: Differences in Reactivation of Tuberculosis Induced from Anti-TNF Treatments Are Based on Bioavailability in Granulomatous Tissue
Source: PLoS Comput Biol. 2007 Oct 19;3(10):e194. doi: 10.1371/journal.pcbi.0030194 (PMC2041971; doi:10.1371/journal.pcbi.0030194)
Supplement: Table S1 — (27 KB DOC) [file pcbi.0030194.st001.doc]

### Table S1

| **Experiment** | **Effect on antibacterial protection** | **Effect on granuloma development** | **Reference** |
| --- | --- | --- | --- |
| TNF KO | increased CFU | delayed formation | 2 |
| TNFR1 KO | increased CFU | delayed formation and necrosis | 4 |
| Anti-TNF during initial infection | increased CFU | delayed formation and necrosis | 4 |
| Anti-TNF in chronic infection | increased CFU | disorganization and diffuse infiltration | 1 |
