# Supplementary material for: Differences in Reactivation of Tuberculosis Induced from Anti-TNF Treatments Are Based on Bioavailability in Granulomatous Tissue
Source: PLoS Comput Biol. 2007 Oct 19;3(10):e194. doi: 10.1371/journal.pcbi.0030194 (PMC2041971; doi:10.1371/journal.pcbi.0030194)
Supplement: Table S2 — (20 KB DOC) [file pcbi.0030194.st002.doc]

Table S2

| **TNF production** | | | |
| --- | --- | --- | --- |
|  |  | *Time post infection* | *Concentration (pg/ml)^(*)^* |
| By Macrophages | | Early (<100 days) | 0.001 - 1 |
|  |  | Late (>100 days) | 0.4 - 1 |
| *By T cells* | | Early (<100 days) | 0.0001 - 0.01 |
|  |  | Late (>100 days) | 0.4 - 1 |

*^(*)^* 5% of these concentrations represents tmTNF, while 95% represents sTNF
