# Supplementary material for: Differences in Reactivation of Tuberculosis Induced from Anti-TNF Treatments Are Based on Bioavailability in Granulomatous Tissue
Source: PLoS Comput Biol. 2007 Oct 19;3(10):e194. doi: 10.1371/journal.pcbi.0030194 (PMC2041971; doi:10.1371/journal.pcbi.0030194)
Supplement: Table S3 — (21 KB DOC) [file pcbi.0030194.st003.doc]

Table S3

|  | **Anti-TNF antibody^60^** | **TNF receptor fusion^61^** |
| --- | --- | --- |
| *Description* | Chimeric monoclonal TNF-antibody | TNF receptor p75-IgG fusion protein rather than an antibody. |
| *Binding* | It binds essentially irreversibly to monomeric and trimeric TNF, both soluble and membrane bound but does not bind to soluble LTα_3_. | It binds selectively to trimeric sTNF and LTα_3_, with a four-fold lower affinity for tmTNF (wrt Infliximab).  Faster dissociation rate: it sheds 50% of sTNF and 90% of mTNF in only 10 minutes |
| *PD-PK* | Half life of 9.5 days | Half life of 3.5 days |
| *Dose and administration* | 2 hr IV infusion  RA: 3-10 mg/Kg at weeks 0, 2, 6  and every 4-8 weeks  CD: 5 mg/kg at weeks 0, 2, 6 | SC injections  RA, PsA, AS: 25 mg biweekly (BIW), 50 mg per week (QW)  JRA: 0.4 mg/kg (BIW), 0.8 mg/kg (QW) |
