# Supplementary material for: Differences in Reactivation of Tuberculosis Induced from Anti-TNF Treatments Are Based on Bioavailability in Granulomatous Tissue
Source: PLoS Comput Biol. 2007 Oct 19;3(10):e194. doi: 10.1371/journal.pcbi.0030194 (PMC2041971; doi:10.1371/journal.pcbi.0030194)
Supplement: Table S4 — (22 KB DOC) [file pcbi.0030194.st004.doc]

Table S4

| **VCT1**  (starts at day 500, 100 runs per each treatment) | | **VCT2**  (starts at day 500, 100 runs per each treatment) | | **VCT3**  (starts at day 0, 12 runs per each treatment) | |
| --- | --- | --- | --- | --- | --- |
| *Parameter varied* | *Range* | *Parameter varied* | *Range* | *Parameter varied* | *Range* |
| *bioav* | 0 -100% | *bioav* | 20 -50% | *bioav* | 0 -50% |
| σ | 50 -100% | σ | 50 -100% | σ | 70 -100% |
| Cell loss^(*)^ | (***) | Cell loss^(*)^ | (***) | Cell loss^(*)^ | (***) |
| TNF-independent recruitment^(**)^ | 0 -100% | TNF-independent recruitment^(**)^ | 20 -50% | TNF-independent recruitment^(**)^ | 0 -100% |
| Bacterial level at treatment initiation | 2e3 | Bacterial level at treatment initiation | 5e2 - 4e3 | Bacterial level at treatment initiation | 25 |
| Duration of treatment | 12 | Duration of treatment | 12 - 24 months | Duration of treatment | 12 |

^(*)^only for anti-TNF Ab treatment. ^(**)^only for TNF receptor fusion treatment. ^(***)^see Supplementary Table 6 for parameter variations
