# Supplementary material for: Differences in Reactivation of Tuberculosis Induced from Anti-TNF Treatments Are Based on Bioavailability in Granulomatous Tissue
Source: PLoS Comput Biol. 2007 Oct 19;3(10):e194. doi: 10.1371/journal.pcbi.0030194 (PMC2041971; doi:10.1371/journal.pcbi.0030194)
Supplement: Table S5 — (21 KB DOC) [file pcbi.0030194.st005.doc]

Table S5

| Parameter | **Description** | **Correlation with bacterial load** | **Significance** |
| --- | --- | --- | --- |
|  |  |  |  |
| μ_tmTNF-MA_ | MA-loss induced by anti-TNF antibody | Positive (0.3) | Significant ^(*)^ only 100 days PTI |
| μ_tmTNF-MI_ | MI-loss induced by anti-TNF antibody | Negative  (-0.5 , -0.75) | Always very significant |
| μ_tmTNF-T8_ | T8-loss induced by anti-TNF antibody | Positive (0.3) | Significant only in the first 100 days PTI |

(*) significant (p<0.05), very significant: p<0.001; PTI= Post Treatment Initiation, MA=activated macrophage, MI=infected macrophage T8=Effector CD8+ T cells
