# Supplementary material for: Differences in Reactivation of Tuberculosis Induced from Anti-TNF Treatments Are Based on Bioavailability in Granulomatous Tissue
Source: PLoS Comput Biol. 2007 Oct 19;3(10):e194. doi: 10.1371/journal.pcbi.0030194 (PMC2041971; doi:10.1371/journal.pcbi.0030194)
Supplement: Table S6 — New Parameter Estimates in addition to those estimated previously [28,31] (shown in parentheses are the values used to generate a latent state, see Figure 1). (101 KB DOC) [file pcbi.0030194.st006.doc]

**Table S6**

| **Parameter** | **Description** | **Range** | | **Reference** | | **Units** |
| --- | --- | --- | --- | --- | --- | --- |
| p | % of sTNF vs tmTNF | 0 - 1 (0.95) | | ^12,41^, estimated | | scalar |
| d | Direct “down-regulation” effect of macrophage activation through tmTNF reverse signaling | 0 - 100 (1) | | Estimated | | scalar |
| s_tmTNF_ | Half sat., effect of tmTNF on T cell activation through tmTNF reverse signaling | 0.1 - 100 (1) | | Estimated | | pg/ml |
| f_3_ | Adjustment, tmTNF on IFN-γ | 0.01 - 100 (2) | | Estimated | | scalar |
| μ_tmTNF-MI_ | Max rate of MI loss induced by the drug (either complement or reverse signaling) | 0.01 -10 | | Estimated | | 1/day |
| μ_tmTNF-MA_ | Max rate of MA loss induced by the drug (either complement or reverse signaling) | 0.01 -10 | | Estimated | | 1/day |
| μ_tmTNF-T1_ | Max rate of T1 loss induced by the drug (either complement or reverse signaling) | 0.01 -10 | | Estimated | | 1/day |
| μ_tmTNF-T2_ | Max rate of T2 loss induced by the drug (either complement or reverse signaling) | 0.01 -10 | | Estimated | | 1/day |
| μ_tmTNF-T8_ | Max rate of T8 loss induced by the drug (either complement or reverse signaling) | 0.01 -10 | | Estimated | | 1/day |
| μ_tmTNF-TC_ | Max rate of TC loss induced by the drug (either complement or reverse signaling) | 0.01 -10 | | Estimated | | 1/day |
| s_tmTNF-MI_ | Half sat., effect of tmTNF on MI loss | 0.01 - 1000 | | Estimated | | MI |
| s_tmTNF-MA_ | Half sat., effect of tmTNF on MA loss | 0.01 - 1000 | | Estimated | | MA |
| s_tmTNF-T1_ | Half sat., effect of tmTNF on T1 loss | 0.01 - 1000 | | Estimated | | T1 |
| s_tmTNF-T2_ | Half sat., effect of tmTNF on T2 loss | 0.01 - 1000 | | Estimated | | T2 |
| s_tmTNF-T8_ | Half sat., effect of tmTNF on T8 loss | 0.01 - 1000 | | Estimated | | T8 |
| s_tmTNF-TC_ | Half sat., effect of tmTNF on TC loss | 0.01 - 1000 | | Estimated | | TC |
| scaling1 | Scaling factor for all TNF-independent recruitment parameters for T cells | 1 – 300 (200) | | Estimated | | scalar |
| scaling2 | Scaling factor for all TNF-dependent recruitment parameters for T cells | 0.01 – 5 (1) | | Estimated | | scalar |
| scaling1M | Scaling factor for all TNF-independent recruitment parameters for macrophages | 0.001 – 0.1 (0.01) | | Estimated | | scalar |
| scaling2M | Scaling factor for all TNF-dependent recruitment parameters for macrophages | 0.001 – 0.1 (0.01) | | Estimated | | scalar |
| **These parameters were estimated previously in ^28,31^**(value in () is used to generate latent state) | | | | | | |
| **Parameter** | **Description** | | **Range** | | **Units** | |
| α_5a_ | IFN-γ production by Th1 | | 1-100 (50) | | pg/Th1 day | |
| α_30_ | TNF Production by MI | | 1e-3 – 2e-2 (3e-3) | | pg/ml MI day | |
| α_5c_ | IFN-γ production by MI | | 0.0002-0.0006 (0.0003) | | pg/ml MI | |
| α_4a_ | TNF-independent recruitment of MR | | 5e-2 | | 1/ day | |
| α_23_ | IL-12 production by MR | | 1e-4 – 0.1 (2e-4) | | pg/ml MR | |
| α_5b_ | IFN-γ production by T8 cells | | 1-100 (50) | | pg/T8 day | |
| α_18_ | IL-10 production by TCs and T8s | | 0.0002 – 0.06 (2e-2) | | pg/(CD8total) day | |
| α_3A2_ | Th2 recruitment by chemokines | | 0.001 | | 1/day | |
| α_3ac_ | TNF-independent recruitment of TC/T8 | | 0.003 | | 1/ day | |
| α_31_ | TNF production by MA | | 0.3e-3 – 1.5e-2 (4e-3) | | pg/ml MA day | |
| α_1a_ | TNF-independent recruitment of Th0 | | 0.003 | | 1/ day | |
| α_32_ | TNF production by Th1 | | 8.16e-4 | | pg/ml Th1 day | |
| α_33_ | TNF production by T8 | | 0.6e-4 - 1.1e-4 (0.5e-4) | | pg/ml T8 day | |
| α_3a_ | TNF-independent recruitment of Th1 | | 0.003 | | 1/ day | |
| Sr_3B2_ | TNF-dependent recruitment of Th2 | | 1e3 | | 1/day | |
| Sr_4b_ | TNF-dependent recruitment of MR | | 2e4 | | MR/ day | |
| Sr_1b_ | TNF-dependent recruitment of Th0 | | 2e5 | | Th0/ day | |
| Sr_3b_ | TNF-dependent recruitment of Th1 | | 2e4 | | Th1/ day | |
| Sr_3bc_ | TNF-dependent recruitment of TC/T8 | | 8e4 | | T/ day | |
| f_9_ | Ratio Adjustment, TNF/IL10 | | 1 - 100 (50) | | Scalar | |
| f_7_ | Effect of IL-10 on IFN-γ -induced Th0 to Th1 | | 1 | | scalar | |
| f_8_ | Ratio Adjustment, IL-10/TNF on MR recruitment | | 1 – 100 (1) | |  | |
| S_4b1_ | Half sat., effect of TNF on Th1 recruitment | | 160 - 200 (165) | | pg/ml | |
| S_4b2_ | Half sat., effect of TNF on Th0 recruitment | | 100 - 500 (450) | | pg/ml | |
| S_4b_ | Half-sat, TNF on MR recruitment | | 138 – 556 (200) | | pg/ml day | |
| Β_2_ | Scaling factor of BT for TNF production by MA | | 1e-3 - 1e-4 (1e-3) | |  | |
| Β | Scaling factor of TNF for MR to MA | | 1e2 - 1e5 (1e2) | | BT /pg | |
| C | Half-sat, IFN-γ on Th1 death | | 1067 - 1173 (1100) | | pg/ml | |
| c_c_ | Half-sat, IFN-γ on TC/T8 death | | 530 - 600 (550) | | pg/ml | |
| C_52_ | Half sat., TC on MI killing | | 10 - 100 (50) | | Tc | |
| c_T1_ | Half. sat., effect of Th1 on TC cytotoxicity | | 1 - 1e4 (10) | | Th1 | |
| C_5a_ | Half sat., MA on IFN-γ by Th1 | | 5e3 - 2e4 (7e3) | | MA/ml | |
| c_T_ | Half-sat, BT on TNF production by Th1/T8 | | 1e3 - 1e4 (1e4) | | BT | |
| C_5b_ | Half sat., MA on IFN-γ by T8 | | 1e3 - 1e6 (7e3) | | MA/ml | |
| C_230_ | Half-sat., BT on IL-12 by dendritic cells | | 1e3 - 1e5 (1e3) | | BT/ml | |
| C_23_ | Half sat., BT on IL-12 by MR | | 1e3 - 5e6 (5e3) | | BT/ml | |
| C_4_ | Half-sat, (TC+Th1)/MI on MI apoptosis | | 20 – 60 (40) | | T/MI | |
| w_3_ | Max. % contribution by Th1 to Fas-FasL apoptosis of MI | | 0.4 | |  | |
| w_2_ | Max. % contribution of MI-produced chemokines to MR recruitment | | 0.15 | |  | |
| w_1_ | Max. % contribution of Th1 to cytotoxicity | | 0.5 | |  | |
| M | % overlap between TC and T8 subsets | | 0.5 – 1 (0.6) | | scalar | |
| μ_Ty_ | IFN-γ -induced apoptosis rate of Th1 | | 1e-5 – 1e-3 (1e-4) | | 1/MA day | |
| μ_Tcy_ | IFN-γ -induced apoptosis rate of TC/T8 | | 1e-5 – 1e-3 (1e-4) | | 1/MA day | |
| μ_T8_ | T8 death rate | | 0.33 | | 1/day | |
| μ_Tc_ | Tc death rate | | 0.33 | | 1/day | |
| μ_T80_ | T80 death rate | | 0.33 | | 1/day | |
| μ_I_ | BI turnover to BE due to MI death, other mechanisms | | 0 - 0.005 (0.004) | | 1/day | |
| μ_TNF_ | TNF decay rate | | 1.112 | | 1/day | |
| k_14a_ | Fas-FasL induced apoptosis of MI | | 0.01 - 0.1 (0.1) | | 1/day | |
| k_14b_ | TNF induced apoptosis of MI | | 0.1 - 0.8 (0.1) | | 1/day | |
| k_52_ | Cytotoxic killing of MI | | 0.07 - 1 (0.5) | | 1/day | |
| s_10_ | Half sat., IFN-γ on TNF production by MA | | 50 - 100 (80) | | pg/ml | |
| s_12_ | Dendritic cell production of IL-12 | | 200 - 1000 (300) | | pg/ml day | |
| S | Describes IL-10 downregulation of IL-12 by MA | | 1 - 100 (10) | | pg/ml | |
| δ_7_ | IL-10 production by MA | | 0.001 - 0.01 (0.01) | | pg/ml MA | |
| N_fraca_ | Avg. number of bacteria within a single MI released upon TNF-induced apoptosis | | 0.4 - 0.8 (0.5) | | scalar | |
| N_fracc_ | Avg. number of bacteria within a single MI released upon Fas-FasL apoptosis | | 0.05 - 0.2 (0.1) | | scalar | |
| α_20_ | BE growth rate | | 0 - 0.26 (0.05) | | 1/day | |
| α_19_ | BI growth rate | | 0.17 - 0.6 (0.4) | | 1/day | |
| α_12_ | IL-4 production by Th2 | | 1e-3 – 9.1e-3 (1e-3) | | pg/Th2 day | |
| α_11_ | IL-4 production by Th0 | | 2.8e-4 – 4e-3 (5e-4) | | pg/Th0 day | |
| α_17_ | IL-10 production by Th2 | | 6e-4 – 6e-2 (6e-2) | | pg/Th2 day | |
| α_8_ | IL-12 production by MA | | 8e-5 | | pg/MA day | |
| α_7_ | IFN-γ production by Th0 | | 0.02 - 0.06 (0.03) | | pg/ml Th0 | |
| α_16_ | IL-10 production by Th1 | | 2e-4 – 1e-3 (2e-3) | | pg/Th1 day | |
| α_2_ | Max. growth rate of Th0 | | 1e-4 - 2.8 (5e-3) | | 1/day | |
| S_rm_ | MR recruitment rate | | 600 - 1000 (1000) | | MR/day | |
| f_6_ | Adjustment, IFN-γ on IL-10 | | 0.025 - 0.053 (0.025) | | scalar | |
| f_4_ | Adjustment, IL-10/IL-12 on IFN-γ | | 0.76 - 3.2 (2) | | scalar | |
| f_2_ | Adjustment, IFN-γ /IL-4 | | 0.0012 - 1.6 (1) | | scalar | |
| f_1_ | Adjustment, IL-4/IFN-γ | | 3 - 410 (200) | | scalar | |
| s_2_ | Half sat., IL-4 | | 1 - 10 (5) | | pg/ml | |
| s_6_ | Half-sat., IL-10 self-inhibition in MA | | 51 - 60 (60) | | pg/ml | |
| s_4_ | Half-sat., IL-12 on IFN | | 50 - 100 (50) | | pg/ml | |
| s_7_ | Half-sat., IL-12 on IFN-γ by NK cells | | 5 – 100 (40) | | pg/ml | |
| s_1_ | Half. sat., IFN-γ on MR to MA | | 50 – 110 (70) | | pg/ml | |
| s_8_ | Half sat., IL-10 on MA deactivation | | 1 – 1000 (1) | | pg/ml | |
| c_9_ | Half. sat, BE on MR infection | | 1e6 - 1e7 (2e6) | | BE | |
| c_8_ | Half. sat., BT on MR activation | | 5e4 - 5e5 (1e5) | | B_T_/ml | |
| c_4_ | Half. sat., T/MI ratio for MI lysis | | 1 – 60 (40) | | T/MI | |
| c_15_ | Half sat., MA on IFN-γ by Th1 | | 1e4 - 5e5 (2e5) | | MA | |
| c_10_ | Half-sat, bacteria on IFN by NK cells | | 1e3 - 1e4 (1e3) | | BT/ml | |
| μ_MR_ | Death rate,MR | | 0.0033 | | 1/day | |
| μ_MI_ | MI death rate | | 0.0011 | | 1/day | |
| μ_MA_ | MA death rate | | 0.07 | | 1/day | |
| μ_Iγ_ | IFN-γ decay rate | | 2.16 - 33.2 (2.16) | | 1/day | |
| μ_I4_ | IL-4 decay rate | | 2.77 | | 1/day | |
| μ_I10_ | IL-10 decay rate | | 3.7 - 7.23 (5) | | 1/day | |
| μ_I12_ | IL-12 decay rate | | 1.188 | | 1/day | |
| μ_T2_ | Th2 death rate | | 0.33 | | 1/day | |
| μ_T1_ | Th1 death rate | | 0.33 | | 1/day | |
| μ_T0_ | Th0 death rate | | 0.33 | | 1/day | |
| k_2_ | MR Infection rate | | 0.2 - 0.4 (0.4) | | 1/day | |
| k_3_ | MR Activation rate | | 0.2 - 0.4 (0.1) | | 1/day | |
| k_17_ | Max. MI death due to BI | | 0.02 - 0.8 (0.02) | | 1/day | |
| k_4_ | MA deactivation by IL-10 | | 0.01 - 0.4 (0.08) | | 1/day | |
| k_6_ | Max. Th0 to Th1 rate | | 2.9e-4 - 5e-3 (5e-3) | | ml/pg day | |
| k_7_ | Max. Th0 to Th2 rate | | 0.02 - 0.7 (0.02) | | ml/pg day | |
| k_18_ | BE killing by MR | | 1.2e-9 – 1.2e-8 (5e-9) | | ml/MR day | |
| k_15_ | BE killing by MA | | 1.25e-7 | | ml/MA day | |
| s_g_ | IFN-γ production by NK cells | | 0 – 1000 (100) | | pg/ml day | |
| N | Max. MOI of MI | | 10 – 100 (20) | | BI/MI | |
