# Supplementary material for: Differences in Reactivation of Tuberculosis Induced from Anti-TNF Treatments Are Based on Bioavailability in Granulomatous Tissue
Source: PLoS Comput Biol. 2007 Oct 19;3(10):e194. doi: 10.1371/journal.pcbi.0030194 (PMC2041971; doi:10.1371/journal.pcbi.0030194)
Supplement: Figure S2 — (130 KB PDF) [file pcbi.0030194.sg002.pdf]

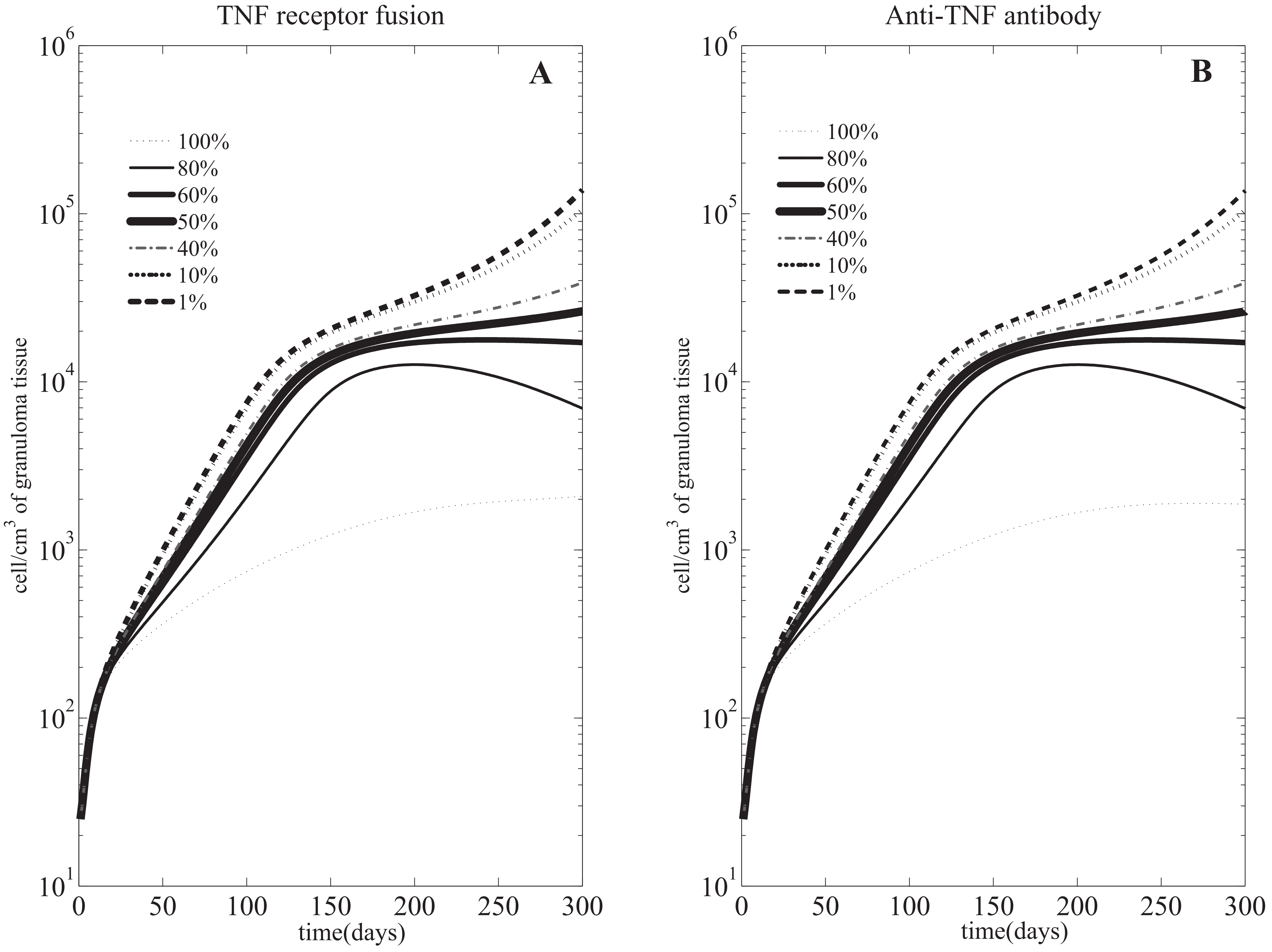
TNF receptor fusion
Anti-TNF antibody
A
B
100%
80%
60%
50%
40%
10%
1%
cell/cm^3 of granuloma tissue
time(days)
10^1
10^2
10^3
10^4
10^5
10^6
0
50
100
150
200
250
300
