# Supplementary material for: Differences in Reactivation of Tuberculosis Induced from Anti-TNF Treatments Are Based on Bioavailability in Granulomatous Tissue
Source: PLoS Comput Biol. 2007 Oct 19;3(10):e194. doi: 10.1371/journal.pcbi.0030194 (PMC2041971; doi:10.1371/journal.pcbi.0030194)
Supplement: Text S1 — (40 KB PDF) [file pcbi.0030194.sd001.pdf]

TEXT S1

TNF Biology

TNF forms and its receptors

All soluble TNF is derived from tmTNF by proteolytic cleavage, but not all tmTNF goes on to be cleaved to generate sTNF[58]. Macrophages produce more sTNF than lymphocytes and the expression and effect of tmTNF is transient considering that it is degraded very fast[41]. Whether this translates to more tmTNF expressed on T cells is not known. Both forms of TNF function by binding to one of two receptors, TNFR1 (TNFRp55) and TNFR2 (TNFRp75)[13]. TNFR1 is constitutively expressed in most tissues, whereas expression of TNFR2 is highly regulated and is typically found in cells of the immune system[4]. The receptor TNFR2 has a lower affinity for sTNF than does TNFR1. Soluble TNF therefore stimulates TNFR1 more efficiently and effectively than TNFR2 (by some estimates as much as 20 fold differential[58]). Trans-membrane TNF stimulates TNFR1 and TNFR2 equally well. Anti-TNF Ab treated wild type mice and TNFR1 KO mice do not survive and are essentially identical in acute infection[4]. There is no evidence that in the absence of TNFR1, TNFR2 would signal through NFκβ and elicit an acceptable inflammatory response. In fact, only TNFR1 KO mice are susceptible to *M. tuberculosis*. Known effects of soluble TNF and tmTNF on macrophages and T cells are summarized in Table 1 (see [62] for a review).

Reverse signaling

Transmembrane TNF also contains a casein kinase I motif and itself can act as a receptor[SN2]. Transmembrane TNF engagement can be triggered both by TNFR positive cells and by TNF specific antibodies[SN3]. Reverse signaling (RS) of tmTNF confers resistance to LPS in monocytes and macrophages[18], causing an anergic state of

monocytic cells. Under pathological conditions tmTNF might additionally transmit apoptotic signals, as has been observed for monocytes and T cells from patients with Crohn's disease[19,SN4]. Trans-membrane TNF stimulation on CD4+ T cells can reversely induce the production of higher amounts of IL-2 and adhesion molecules, such as E-selectin[SN5,SN6,16].

Clearly more experimental evidence is needed to validate whether reverse signaling of TNF ligand family members is important *in vivo*. Bi-directional signaling requires direct cell-to-cell contact and could be relevant in situations where cells cluster, such as granulomas. The effect of reverse signaling so far described in B and T cells is of a stimulatory nature[16,17], whereas monocytes are mainly inhibited in their effector functions[18,19,20,SN7]. Based on the literature, the overall effects of reverse signaling by tmTNF seem to be minimal in the absence of anti-TNF treatment. Under pathological conditions (chronic inflammatory states), the presence of anti-TNF antibodies (and not TNF-receptor fusion molecules) and the consequent binding to tmTNF can induce:

i) Activation of the complement cascade (due to high concentration of Abs)[SN8]

ii) Apoptosis induced by RS through tmTNF binding[19,SN4]

Activation of complement cascade is supported by data on Crohn's disease and it might not be a mechanism shared among all the TNF-related pathologies (such as Rheumatoid Arthritis and Ankylosing Spondylitis). The likely consequence of triggering the complement cascade is the release of intracellular bacteria, while apoptosis kills most of the intracellular bacteria[SN9,53].

The role of lymphotoxin in M. tuberculosis infection

Lymphotoxin (LT) comprises two members of the TNF superfamily, LTα and LTß. LT is active as a secreted homotrimeric molecule (LT_3, also known as TNF-ß)[SN10], which binds

to both of the TNF receptors (TNFR1 and TNFR2)[SN11,SN12]. LTα_3 is produced mainly by $CD4^+$ T cells, B cells, and NK cells[SN13]. As LTα_3 and TNF bind to TNFR1 with similar affinity[SN14] and have 30% homology in amino acid sequence[SN15], the functional activity for LTα_3 independent of TNF is incompletely understood. Inhibition of LTα_3 and LTβ has been shown to result in delayed granuloma formation, structural lymph node defects[SN16], delayed macrophage activation and increased bacterial load[SN17], indicating that the TNF superfamily plays a pivotal and non-redundant role in containing infection.

The main effect described in the literature regarding LTα KO mice is abnormal granuloma formation[SN16], with similar numbers of T cells recruited in the lungs but with different localization than in wild type mice[SN16]. In fact, LTα_3 KO chimeras recruited normal numbers of T cells into their lungs, but the lymphocytes were restricted to perivascular and peribronchial areas and were not colocalized with macrophages in granulomas**.** That makes those mice more susceptible. LTα alone cannot rescue TNF KO mice[2]. A critical role for LTα lies not in the activation of T cells and macrophages per se but in the local organization of the granulomatous response.

TEXT S1 REFERENCES

2. Bean, A.G. et al. Structural deficiencies in granuloma formation in TNF gene-targeted mice underlie the heightened susceptibility to aerosol Mycobacterium tuberculosis infection, which is not compensated for by lymphotoxin. *J Immunol* **162**, 3504-11 (1999).
4. Flynn, J.L. et al. Tumor necrosis factor-alpha is required in the protective immune response against Mycobacterium tuberculosis in mice. *Immunity* **2**, 561-72 (1995).
13. Watts, T.H. TNF/TNFR family members in costimulation of T cell responses. *Annu Rev Immunol* **23**, 23-68 (2005).
16. Harashima, S. et al. Outside-to-inside signal through the membrane TNF-alpha induces E-selectin (CD62E) expression on activated human CD4+ T cells. *J Immunol* **166**, 130-6 (2001).
17. Suzuki, I. & Fink, P.J. Maximal proliferation of cytotoxic T lymphocytes requires reverse signaling through Fas ligand. *J Exp Med* **187**, 123-8 (1998).

18. Eissner, G. et al. Reverse signaling through transmembrane TNF confers resistance to lipopolysaccharide in human monocytes and macrophages. *J Immunol* **164**, 6193-8 (2000).
19. Lugering, A. et al. Infliximab induces apoptosis in monocytes from patients with chronic active Crohn's disease by using a caspase-dependent pathway. *Gastroenterology* **121**, 1145-57 (2001).
20. Kirchner, S., Holler, E., Haffner, S., Andreesen, R. & Eissner, G. Effect of different tumor necrosis factor (TNF) reactive agents on reverse signaling of membrane integrated TNF in monocytes. *Cytokine* **28**, 67-74 (2004).
41. Newton, R.C. et al. Biology of TACE inhibition. *Ann Rheum Dis* **60 Suppl 3**, iii25-32 (2001).
53. Oddo, M. et al. Fas ligand-induced apoptosis of infected human macrophages reduces the viability of intracellular Mycobacterium tuberculosis. *J Immunol* **160**, 5448-54 (1998).
58. Kast, R.E. Evidence of a mechanism by which etanercept increased TNF-alpha in multiple myeloma: New insights into the biology of TNF-alpha giving new treatment opportunities-the role of bupropion. *Leuk Res* (2005).
62. Hehlgans, T. & Pfeffer, K. The intriguing biology of the tumour necrosis factor/tumour necrosis factor receptor superfamily: players, rules and the games. *Immunology* **115**, 1-20 (2005).

SN1. Wajant, H., Pfizenmaier, K. & Scheurich, P. Tumor necrosis factor signaling. *Cell Death Differ* **10**, 45-65 (2003).

SN2. Watts, A.D. et al. A casein kinase I motif present in the cytoplasmic domain of members of the tumour necrosis factor ligand family is implicated in 'reverse signalling'. *Embo J* **18**, 2119-26 (1999).

SN3. Eissner, G., Kolch, W. & Scheurich, P. Ligands working as receptors: reverse signaling by members of the TNF superfamily enhance the plasticity of the immune system. *Cytokine Growth Factor Rev* **15**, 353-66 (2004).

SN4. van Deventer, S.J. Anti-tumour necrosis factor therapy in Crohn's disease: where are we now? *Gut* **51**, 362-3 (2002).

SN5. Aversa, G., Punnonen, J. & de Vries, J.E. The 26-kD transmembrane form of tumor necrosis factor alpha on activated CD4+ T cell clones provides a costimulatory signal for human B cell activation. *J Exp Med* **177**, 1575-85 (1993).

SN6. Higuchi, M. et al. Membrane tumor necrosis factor-alpha (TNF-alpha) expressed on HTLV-I-infected T cells mediates a costimulatory signal for B cell activation--characterization of membrane TNF-alpha. *Clin Immunol Immunopathol* **82**, 133-40 (1997).

SN7. Kirchner, S. et al. LPS resistance in monocytic cells caused by reverse signaling through transmembrane TNF (mTNF) is mediated by the MAPK/ERK pathway. *J Leukoc Biol* **75**, 324-31 (2004).

SN8. Scallon, B.J., Moore, M.A., Trinh, H., Knight, D.M. & Ghrayeb, J. Chimeric anti-TNF-alpha monoclonal antibody cA2 binds recombinant transmembrane TNF-alpha and activates immune effector functions. *Cytokine* **7**, 251-9 (1995).

SN9. Lammas, D.A. et al. ATP-induced killing of mycobacteria by human macrophages is mediated by purinergic P2Z(P2X7) receptors. *Immunity* **7**, 433-44 (1997).

SN10. Korner, H. & Sedgwick, J.D. Tumour necrosis factor and lymphotoxin: molecular aspects and role in tissue-specific autoimmunity. *Immunol Cell Biol* **74**, 465-72 (1996).
SN11. Hochman, P.S., Majeau, G.R., Mackay, F. & Browning, J.L. Proinflammatory responses are efficiently induced by homotrimeric but not heterotrimeric lymphotoxin ligands. *J Inflamm* **46**, 220-34 (1995).
SN12. Bazzoni, F. & Beutler, B. The tumor necrosis factor ligand and receptor families. *N Engl J Med* **334**, 1717-25 (1996).
SN13. Ware, C.F., Crowe, P.D., Grayson, M.H., Androlewicz, M.J. & Browning, J.L. Expression of surface lymphotoxin and tumor necrosis factor on activated T, B, and natural killer cells. *J Immunol* **149**, 3881-8 (1992).
SN14. Loetscher, H. et al. Recombinant 55-kDa tumor necrosis factor (TNF) receptor. Stoichiometry of binding to TNF alpha and TNF beta and inhibition of TNF activity. *J Biol Chem* **266**, 18324-9 (1991).
SN15. Pennica, D. et al. Human tumour necrosis factor: precursor structure, expression and homology to lymphotoxin. *Nature* **312**, 724-9 (1984).
SN16. Roach, D.R. et al. Secreted lymphotoxin-alpha is essential for the control of an intracellular bacterial infection. *J Exp Med* **193**, 239-46 (2001).
SN17. Ehlers, S. Role of tumour necrosis factor (TNF) in host defence against tuberculosis: implications for immunotherapies targeting TNF. *Ann Rheum Dis* **62 Suppl 2**, ii37-42 (2003).
